# IRF4 contributes to chemoresistance in IGH::BCL2‐positive diffuse large B‐cell lymphomas by mediating BCL2‐induced SOX9 expression

**DOI:** 10.1002/ctm2.70336

**Published:** 2025-05-12

**Authors:** Yirong Zhang, Zizhen Xu, Ruixin Sun, Yixuan Gao, Innocent Agida, Kasimujiang Aximujiang, Lin Yuan, Jiao Ma

**Affiliations:** ^1^ Department of Biochemistry and Molecular Cell Biology Shanghai Jiao Tong University School of Medicine Shanghai PR China; ^2^ Department of Laboratory Medicine College of Health Science and Technology Ruijin Hospital Shanghai Jiao Tong University School of Medicine Shanghai PR China; ^3^ Department of Biochemistry and Molecular Biology School of Basic Medical Sciences Xinjiang Medical University Urumqi PR China; ^4^ Department of Pathology Shanghai General Hospital Shanghai Jiao Tong University School of Medicine Shanghai PR China

**Keywords:** BCL2, chemoresistance, IGH::BCL2‐positive DLBCL, IRF4, SOX9

## Abstract

**Background:**

Diffuse large B‐cell lymphoma (DLBCL), an aggressive type of non‐Hodgkin's lymphoma, has a high relapse/refractory rate. We previously identified sex‐determining region Y (SRY)‐box transcription factor (SOX9) as a transcription factor that serves as a prognostic biomarker, particularly in BCL2‐overexpressing DLBCL, and plays a vital role in lymphomagenesis. However, the molecular mechanisms that modulate the aberrant expression of SOX9 in this DLBCL subset remain unknown.

**Methods:**

Cell viability, apoptosis and cell cycle assays were performed to determine whether SOX9 contributes to DLBCL chemoresistance and rescues silencing IRF4‐induced phenotypes. Protein‒protein interactions and protein ubiquitination were elucidated using immunoprecipitation, immunohistochemistry, immunofluorescence and immunoblotting. Chromatin immunoprecipitation sequencing (ChIP‐seq), ChIP and dual‐luciferase reporter assays were used to investigate IRF4 binding to the SOX9 promoter. The therapeutic potential of IRF4 inhibition was evaluated in vitro and in a mouse model of DLBCL xenografts.

**Results:**

SOX9 enhanced the resistance of the BCL2‐overexpressing DLBCL subset to chemotherapy or a BCL2 inhibitor. Moreover, BCL2 inhibition downregulated SOX9 in an immunoglobulin heavy chain/BCL2‐positive DLBCL subset. We further identified IRF4 as a key regulator of BCL2‐induced SOX9 expression, and ChIP‐seq confirmed that IRF4 is a key transcription factor for SOX9 in DLBCL. In addition, BCL2 promotes IRF4 entry into the nucleus by enhancing protein stability and downregulating proteasomal ubiquitination, thereby enforcing SOX9‐mediated phenotypes. Finally, in a DLBCL cell line and xenografted mouse model, in vivo inhibition of IRF4 with an hIRF4 antisense oligonucleotide repressed lymphomagenesis and DLBCL chemoresistance.

**Conclusions:**

Our data support the conclusion that IRF4 plays an essential role in BCL2‐induced upregulation of SOX9 expression, and targeting IRF4 may represent a promising therapeutic strategy to cure relapsed and refractory DLBCL.

**Keypoints/Highlights:**

- BCL2 activated IRF4 by enhancing its nuclear activity to induce sex‐determining region Y (SRY)‐box 9 protein (SOX9) aberrant expression, which is a critical pathway for drug resistance in BCL2‐overexpressing diffuse large B‐cell lymphoma (DLBCL).
- Targeting IRF4 may be worth investigating further regarding its potential to overcome the chemoresistance of BCL2‐overexpressing DLBCL to standard therapies.

## Graphical abstract

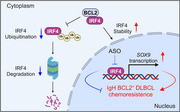

## 1 BACKGROUND

Diffuse large B‐cell lymphoma (DLBCL) is the most common type (30%‒40%) of B‐cell non‐Hodgkin lymphoma, with high death and relapse rates worldwide.^1^ The gold standard induction regimen is a combination of chemotherapeutic agents and monoclonal antibodies known as R‐CHOP.^2^ Although approximately 60% of patients receiving such treatment achieve complete remission, 30%–40% show a poor response,^3^, ^4^ resulting in relapse/refractory disease within 5 years,^5^ shortening median survival to 6.3 months.^6^ The high economic burden and physical and emotional impact of DLBCL make overcoming chemoresistance the primary goal for improving therapeutic efficacy and survival.

Whole‐genome sequencing has uncovered over 300 gene mutations in DLBCL,^7^ especially driver mutations initiating lymphomagenesis and promoting chemoimmunotherapy resistance, including chromosome translocation‐induced overexpression of oncogenes (BCL2, c‐Myc or BCL6),^8^ nuclear factor‐kappa B (NF‐κB) signalling pathway activation by CARD11, MyD88 CD79A/B mutations,^9^ or epigenetic reprogramming by EZH2, MLL2 and CREBBP mutations.^10^ Driver mutation heterogeneity triggers innate or acquired chemoresistance due to sustained exposure to external stress or a modified tumour microenvironment,^11^ which induces expansion of minimal residual tumour clones.^12^ The *IGH::BCL2* translocation t(14;18)(q32;q21) is one of the most common types of cytogenetic abnormalities in lymphoid malignancy, in which, BCL2 gene is translocated to IGH enhancer, resulting in deregulated BCL2 expression.^13^ In addition, *IGH::BCL2* translocation is the hallmark of follicular lymphoma, an indolent subtype. However, approximately 30%–40% of these cases undergo histological transformation to an aggressive lymphoma type such as DLBCL.^14^ International multicentre, randomised phase III clinical trials have shown that BCL2 overexpression or c‐Myc and BCL2 co‐overexpression (double hits)^15^ was significantly associated with poor clinical outcomes and high relapse/refractory rates in patients with DLBCL and was an independent prognostic biomarker for evaluating overall and progression‐free survival of patients with DLBCL following R‐CHOP treatment.^16^

BCL2 is a master regulator of mitochondrial apoptosis, suppressing apoptosis by controlling outer mitochondrial membrane permeabilisation. Several studies have reported that BCL2 exerts its anti‐apoptotic effect by regulating various transcription factors via protein‒protein interactions in a caspase‐independent manner.^17^ BCL2 promotes NF‐κB nuclear translocation and activities of its downstream anti‐apoptotic target molecules.^18^ BCL2 affects Rb phosphorylation, thus inhibiting CDK2 enzymatic activity, which subsequently degrades the phosphorylated Rb protein and inhibits E2F1 transcription activity.^19^ E2F1 binds to the BINP3 promoter, which interacts with BCL2.^20^ These data indicate that BCL2 modulates gene transcription in a complex manner. However, the molecular mechanisms underlying BCL2‐mediated gene transcription remain unclear.

The sex‐determining region Y (SRY)‐box 9 protein (SOX9) belongs to the SOX family of transcription factors that possess high‐mobility‐group box DNA‐binding and transactivation domains.^21^ SOX9 has been implicated in the pathogenesis of various solid tumours, including breast,^22^ cervical,^23^ colorectal^24^ and lung^25^ cancers. Our previous findings showed that SOX9 was preferentially highly expressed in the germinal centre B‐cell‐like (GCB) DLBCL subtype harbouring an IGH/BCL2 translocation and serves as a biomarker that is significantly associated with poor prognosis and response to chemotherapeutics; however, the regulatory mechanism of SOX9 overexpression in this subset remains elusive.

In the present study, we identified IRF4, a master player in plasma cell differentiation,^26^ immune cell proliferation and cell survival,^27^ as a novel transcription factor for SOX9, demonstrating an association between SOX9 induction and BCL2 rearrangement in a small DLBCL subset. We also demonstrated that targeting IRF4 showed promising clinical efficacy in prohibiting lymphomagenesis.

## 2 METHODS

### 2.1 Cell lines and culture conditions

DLBCL cell lines OCI‐LY1, OCI‐LY3 and SUDHL2 were cultured in Iscove's modified Dulbecco's medium supplemented with 20% foetal bovine serum (FBS) and 100 µg/mL penicillin/streptomycin. U2932, SUDHL6, SUDHL8, Karpas‐422 and DB cells were cultured in Roswell Park Memorial Institute 1640 supplemented with 20% FBS and 100 µg/mL penicillin/streptomycin. Cells were maintained in a humidified 37°C/5% CO_2_ incubator. SUDHL2 (CRL‐2956), SUDHL6 (CRL‐2959), SUDHL8 (CRL‐2961) and DB (CRL‐2289) cells were obtained from the American Type Culture Collection. OCI‐LY1 (ACC 722), OCI‐LY3 (ACC 761), U2932 (ACC 633) and Karpas‐422 (ACC 32) cells were obtained from Leibniz Institute DSMZ‐German Collection of Microorganisms and Cell Cultures GmbH cell lines. Cell lines were authenticated by Biowing Biotechnology, and all cells were routinely checked for the mycoplasma test using MycGuard Plus‐Color One‐Step Mycoplasma Detection Kit (catalog no. 40612ES25; Yeasen).

### 2.2 Drug compounds and antibodies

Doxorubicin (catalog no. HY‐15142), ABT‐199 (catalog no. HY‐15531), ABT‐737 (catalog no. HY‐50907), cyclophosphamide (catalog no. HY‐17420), vincristine (catalog no. HY‐N0488A), prednisone (catalog no. HY‐B0214) and rituximab (catalog no. HY‐P9913) were purchased from MedChem Express (Monmouth Junction). The stock solution of CHOP was prepared individually and then mixed‐together according to the ratios of the in vivo treatment (cyclophosphamide:doxorubicin:vincristine:prednisone ≈ 1:.03:.0005:.04), the molar ratio was further converted to µg/mL prior to add to DLBCL cells (please see Table S3 for various drug combination regimens). Control antisense oligonucleotides (ASOs), hIRF4#1‐ASO, hIRF4#2‐ASO and hIRF4#3‐ASO, were designed and synthesised by SYNBIO Technologies. All compounds, except those used for in vivo studies, were reconstituted in dimethylsulphoxide (DMSO) (catalog no. D26563), stored at 100 mM stock concentrations in ‒80°C, and used at the indicated doses as suggested by the vendor. To reconstitute the BCL2 inhibitors, 10 mg ABT‐199 or ABT‐737 powder was dissolved in 115.15 or 122.93 µL DMSO, vortexed to mix, and sonicated to assist solubilisation to obtain a 100 mM stock solution. The immunoblotting antibodies BCL2 (catalog no. sc‐7382) and ATP5A (catalog no. sc‐136178) were purchased from Santa Cruz Biotechnology, SOX9 antibody (catalog no. AB5535) from Millipore, IRF4 (catalog no. 62834) antibody from Cell Signaling Technology and Lamin B (catalog no. 12987‐1‐AP), β‐tubulin (catalog no. 12987‐1‐AP) and GAPDH (catalog no. 60008‐1‐Ig) were obtained from Proteintech.

### 2.3 Cell proliferation assay

Cell proliferation was analysed using Cell Counting Kit‐8 (CCK‐8; Yeasen). Briefly, the cells were seeded in 96‐well plates at 5000 cells/well. Ten microlitres of CCK‐8 was added to each well at 24, 48 or 72 h and incubated at 37°C for 4 h. Subsequently, absorbance was measured at 450 nm using a microplate reader.

### 2.4 Annexin V APC/7AAD apoptosis detection assay

DLBCL cells were treated either with ABT‐199, ABT‐737 at the indicated doses or R‐CHOP (CHOP plus 10 µg/mL Rituximab) for 48 h. Apoptosis was detected using the Annexin V‐APC Apoptosis Detection Kit II (BD Biosciences) according to the manufacturer's protocol.

### 2.5 Cell cycle assay

The cell cycle assay was performed using the APC BrDU Kit (BD Biosciences) according to the manufacturer's instructions.

### 2.6 RNA isolation and real‐time polymerase chain reaction

Total RNA from mouse tumour tissues and cell samples was extracted using TRIzol reagent (Tiangen). A NanoDrop spectrophotometer was used to measure RNA quality and quantity. One microgram of total RNA was reverse transcribed using FastKing gDNA Dispelling RT SuperMix (Tiangen). Real‐time analysis was performed using SYBR Green Master Mix with sequence‐specific predesigned primers (Takara). For quantitative analysis, target gene values were normalised to GAPDH or 18S gene expression using the ΔΔCT method. Primer sequences are listed in Supporting Information.

### 2.7 Chromatin immunoprecipitation sequencing and data processing

A sequencing library was prepared by converting 10 ng DNA from each sample into a phosphorylated blunt‐end fragment. An ‘A’ base was added to the 3′ end of the blunt phosphorylated DNA fragments, and Illumina's genomic adapters were ligated to the A‐tailed DNA fragments before polymerase chain reaction (PCR) amplification to enrich ligated fragments. An enriched product of approximately 200–1500 base pairs was selected using AMPure XP beads. The library was denatured with.1 M NaOH to generate single‐stranded DNA molecules, loaded onto channels of the flow cell at 8 pM concentration, and amplified in situ using a Cluster Kit. Sequencing was carried out by running 150 cycles twice on the Illumina NovaSeq 6000 (Illumina) system according to the manufacturer's instructions. After the sequencing images were generated, image analysis and base calling were performed using Off‐Line Basecaller software (OLB V1.8). Sequence quality was examined using FastQC software. After passing through a Solexa Chastity quality filter (Illumina) clean reads were aligned to the human reference genome UCSC HG19 using BOWTIE (V2.1.0). Aligned reads were used for peak calling of chromatin immunoprecipitation (ChIP) regions using MACS V1.4.2. Statistically significant ChIP‐enriched regions (peaks) were identified by comparing immunoprecipitation versus input or a Poisson background model, using a *p*‐value threshold of 10^−3^. Peaks were annotated to the nearest gene using the latest UCSC RefSeq database.

### 2.8 ChIP assay

The Pierce Agarose ChIP Kit was purchased from Cell Signaling Technology. The cells were crosslinked with 1% formaldehyde for 10 min at room temperature and the reaction terminated by adding glycine (1.25 M). Fixed cells were harvested in sodium dodecyl sulphate buffer with a protease inhibitor and then sonicated to generate DNA fragments of 200–1000 base pairs. The sheared chromatin‐lysed extracts were incubated overnight at 4°C with anti‐IRF4 antibody or control immunoglobulin G with rotation. After immunoprecipitation of the cross‐linked protein and DNA, the immunocomplexes were reverse transcribed to free DNA. PCR was performed using input DNA or immunoprecipitated products. The PCR products were separated by agarose gel electrophoresis.

### 2.9 Co‐immunoprecipitation

Co‐immunoprecipitation was performed as previously described.^28^

### 2.10 Plasmid construction and dual‐luciferase reporter assay

SOX9 promoter‐luciferase reporter plasmids containing the IRF4 promoter region were constructed using the pGL3 plasmid. Wild‐type and mutant IRF4 promoter‐luciferase constructs were verified by DNA sequencing. Dual‐luciferase reporter assay (Promega) was performed according to the manufacturer's instructions.

### 2.11 Ubiquitination assay

This assay was performed as described previously.^28^

### 2.12 Immunofluorescence

We determined the subcellular localisation of BCL2 and IRF4 proteins in DLBCL cell lines. OCI‐LY1, OCI‐LY3 and SUDHL6 cells were fixed in 4% formaldehyde for 10 min at room temperature and washed three times with ice‐cold phosphate‐buffered saline (PBS). The cells were permeabilised with.2% Triton X‐100 for 10 min at room temperature. Fixed cells were pre‐incubated in blocking solution (10% bovine serum albumin in PBS), followed by incubation with primary antibodies at 4°C overnight. After incubation with IRF4 anti‐rabbit or BCL2 anti‐mouse primary antibodies, cells were washed three times with shaking in PBS and probed with fluorescence‐conjugated anti‐rabbit or anti‐mouse secondary antibodies (Alexa Fluor 594 or 488). After washing three times with PBS, DAPI was used for DNA counterstaining, followed by mounting on slides. Fluorescence images were obtained using a Zeiss 880 spectral confocal laser‐scanning microscope (Oberkochen) on the SJTU Microscopy Service platform.

### 2.13 Immunohistochemistry

After incubation at 60°C for 4 h, paraffin sections were dewaxed and hydrated and then boiled with citrate buffer (pH 6.0) for antigen retrieval. Endogenous peroxidase activity was blocked by incubation in hydrogen peroxide (3%) for 10 min. The sections were treated for 30 min with 5% goat serum before incubating with anti‐SOX9, anti‐BCL2 or anti‐IRF4 primary antibodies overnight at 4°C. The sections were then stained with diaminobenzidine after incubation with a secondary antibody for 1 h. Two pathologists performed a double‐blinded examination to evaluate the immunostaining of SOX9, BCL2 and IRF4. A scoring system was created as previously described.^29^

### 2.14 Animal studies

#### 2.14.1 DLBCL xenografts

All experiments were performed using an Institutional Animal Care and Use Committee‐approved protocol, and institutional guidelines for the proper use of animals in research were followed. Six‐week‐old female nude mice were purchased from Shanghai Ling Chang Biotechnology Co. with the approval of the Institutional Animal Facility at Shanghai Jiao Tong University Medical School. SUDHL8‐shIRF4 and parental SUDHL8 xenografts were established by injecting approximately 5 million cells in 50% Matrigel into the flanks of the mice.

#### 2.14.2 Drug administration and synergy

Mice bearing established xenografts (tumour volume, 100–200 mm^3^) were randomised into control or drug treatment groups and treated with or without intraperitoneal injection of 2.5 mg/kg doxorubicin every 2 days (four times), 25 mg/kg ABT‐199 with gastric gavage every 2 days (three times), intraperitoneal injection of 25 mg/kg hIRF4‐ASO#3 three times a week for 3 weeks, or combinations of these treatments at the indicated time intervals. Tumours were measured three times per week using a caliper. Tumour volumes were calculated as length × width × width/2. The fold change in tumour volume was determined by normalising each tumour measurement to its volume on day 0 at the beginning of drug administration.

### 2.15 Statistical analyses

A two‐sided Student's *t*‐test was used to compare differences between two groups. Data are presented as means ± SD and *p* < .05 was considered statistically significant. Two‐way analysis of variance was used for comparing assays involving at least three groups. Correlations between BCL2 and SOX9 immunochemical staining positivity in DLBCL patient samples were determined using simple linear regression analysis. All statistical tests were two‐sided, with an alpha level of.05 as the significance cutoff. All analyses were performed using SAS version 9.4 (SAS Institute), and graphs were generated using GraphPad software version 10.1.

## 3 RESULTS

### 3.1 Silencing BCL2‐suppressed SOX9 expression in BCL2‐overexpressing DLBCL

**FIGURE 1:**
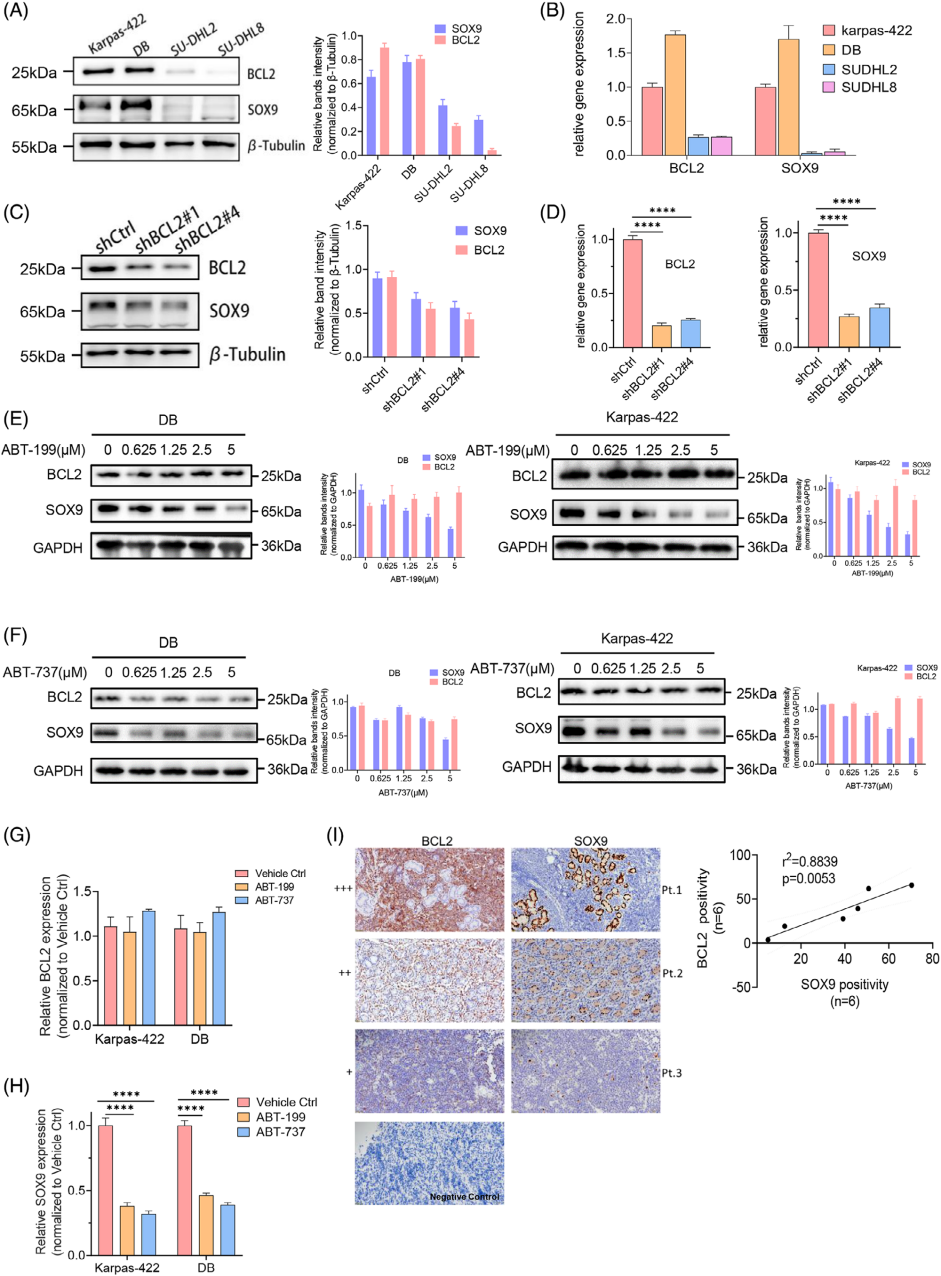
Silencing BCL2 suppresses sex‐determining region Y (SRY)‐box 9 protein (SOX9) expression in BCL2 overexpressed germinal centre B‐cell‐like (GCB) diffuse large B‐cell lymphoma (DLBCL) subsets. (A and B) Immunoblotting and real‐time polymerase chain reaction (PCR) assay of BCL2 and SOX9 protein and mRNA levels in a panel of DLBCL cell lines (Karpas‐422, DB, SUDHL2 and SUDHL8), DB cells were transduced either with lentiviral encoding scramble control, shBCL2#1 or shBCL2#2 plasmids for 72 h, respectively, prior to subject flow cytometry sorting of Green fluorescent protein (GFP)‐positive cells to generate stable transfectants. (C and D) Immunoblotting or real‐time PCR assays of protein or mRNA level of BCL2 and SOX9. Two‐way analysis of variance (ANOVA) was used to compare scramble Ctrl to shSOX9#1 and shSOX9#2. (E and F) Immunoblotting assay of BCL2 and SOX9 protein levels in Karpas‐422 and DB cells were either treated with indicated doses of ABT‐737 or ABT‐199 for 48 h. (G and H) Real‐time PCR of BCL2 and SOX9 mRNA levels in Karpas‐422 and DB cells were either treated with 2.5 µM ABT‐737 or ABT‐199 for 48 h. Two‐way ANOVA was performed to compare vehicle Ctrl to treated. (I) BCL2 and SOX9 immunostainings were denoted as (+), (++) and (+++) to indicate the expression levels of BCL2 and SOX9. Negative control was included to eliminate the false‐positive staining. Positivity of BCL2 or SOX9 immunostaining was measured using ImageJ software, the simple linear regression statistical analysis was carried out to determine the association between SOX9 and BCL2 positives (*n* = 6, 200× magnification). β‐Tubulin or GAPDH was included as indications of equal loading. Protein levels were quantified (normalised to housekeeping genes) using ImageJ software and graph was generated using GraphPad version 9.0. All experiments were repeated three times, and graph with error bars show the data represent the mean ± standard deviation (SD) from technical triplicates (^****^ *p* < .005).

Although our previous study revealed the oncogenic role of SOX9 in a subset of *IGH::BCL2* translocated GCB DLBCL, the mechanism underlying the association between SOX9 overexpression and BCL2 rearrangement is unclear. As *IGH::BCL2* translocation occurs in approximately 20%–30% of patients with de novo DLBCL and results in BCL2 overexpression, which is associated with a poor prognosis of DLBCL, we investigated whether BCL2 induces SOX9 expression in specific DLBCL subsets. Endogenous BCL2 and SOX9 expression was detected using immunoblotting and quantitative PCR in DLBCL cell lines. BCL2 and SOX9 protein and mRNA levels were high in t(14;18)‐positive Karpas‐422 (GCB) and DB cells (GCB), whereas t(14;18)‐negative SUDHL2 (ABC) and SUDHL8 cells (GCB), displayed low BCL2 and SOX9 expression levels (Figure 1A,B). BCL2 silencing downregulated SOX9 protein and mRNA levels (Figure 1C,D), suggesting that SOX9 expression was associated with BCL2. To further explore whether suppressing BCL2 activity modulated SOX9 expression in DLBCL. Karpas‐422 and DB cells were treated with.625, 1.25, 2.5 or 5 µM ABT‐199 or ABT‐737. The selective targeting of BCL2 by ABT‐199 or ABT‐737 downregulated SOX9 expression (Figure 1E‒H). Both BCL2 inhibitors induced apoptosis without a significant impact on BCL2 protein levels, as reported previously^30^ (Figures 1G and S1). Collectively, our results suggested that SOX9 was highly expressed in BCL2‐overexpressing DLBCL cells. To further validate whether SOX9 expression correlates with that of BCL2, tumour tissues from six DLBCL cases were subjected to immunohistochemistry to determine the BCL2 and SOX9 expression levels. As shown in Figure 1I, BCL2 and SOX9 expression levels were significantly correlated with each other (*p* = .0053; see Table S1 for the patient's clinical information).

### 3.2 SOX9 reduced drug sensitivity of BCL2‐overexpressing DLBCL

**FIGURE 2:**
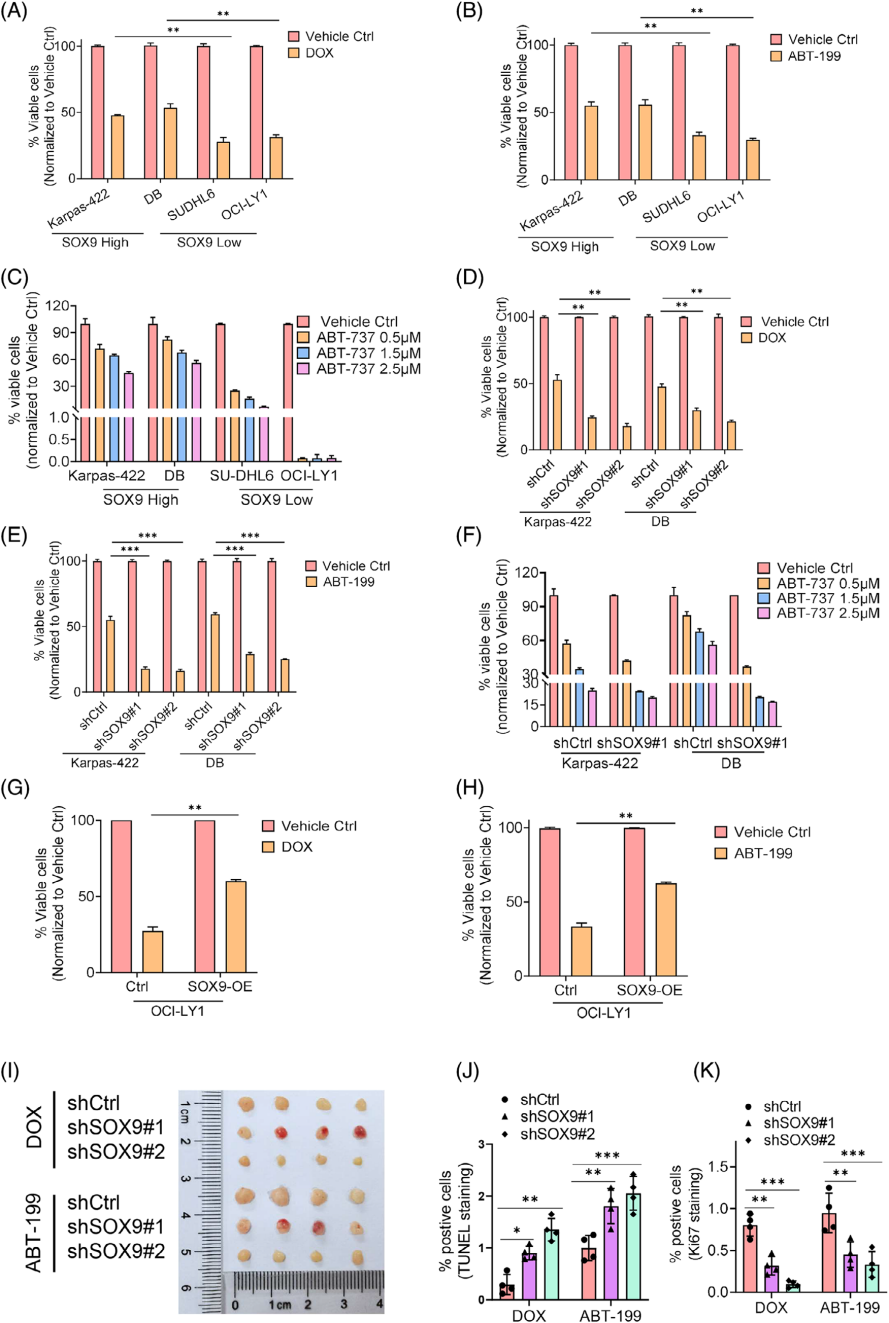
Sex‐determining region Y (SRY)‐box 9 protein (SOX9) reduced drug sensitivity of BCL2 overexpressed germinal centre B‐cell‐like (GCB) diffuse large B‐cell lymphoma (DLBCL) subsets. (A‒C) Karpas‐422, DB (SOX9 high expressing cells) or SUDHL6, OCI‐LY1 (SOX9 low expressing cells) were treated either with vehicle control, 2.5 µM doxorubicin, 2.5 µM ABT‐199 or various doses of ABT‐737 (.5, 1.5 and 2.5 µM) for 48 h, respectively, followed by flow cytometry to determine the percentage of viable cells. Two‐way analysis of variance (ANOVA) was performed to compare the difference of viable cells (%) between SOX9 high expressing cells and SOX9 low expressing cells upon drug treatment. (D‒F) Karpas‐422 or DB cells were transduced either with scramble control, shSOX9#1 or shSOX9#2 lentiviral plasmids for 72 h, followed by flow cytometry sorting out GFP‐positive cells to obtain stable transfectants. Stable cells were then treated either with vehicle control, 2.5 µM DOX, 2.5 µM ABT‐199 or ABT‐737 (.5, 1.5 and 2.5 µM) for 48 h, followed by flow cytometry to determine the percentage of viable cells. Two‐way ANOVA was performed to compare the difference of viable cells (%) between scramble Ctrl and SOX9 silencing groups. (G and H) OCI‐LY1 cells were transduced either with empty vector or PCDH‐SOX9 overexpression lentiviral plasmids for 48 h followed by flow sorting of GFP‐positive cells to obtain stable transfectants. SOX9‐overexpressing OCI‐LY1 stable transfectants were then either with vehicle control, 2.5 µM doxorubicin or 2.5 µM ABT‐199 for 48 h, followed by flow cytometry to determine the percentage of viable cells. One‐way ANOVA was performed to compare the difference between empty vector and SOX9 overexpression groups. (I) Ten millions of lentiviral encoding scramble control, shSOX9#1 and shSOX9#2 plasmids transducing BD cells were engrafted to generate DLBCL xenografts via subcutaneous injection prior to administrate mice either with vehicle control or DOX (2.5 mg/kg) every 2 days for four times or ABT‐199 (25 mg/kg) very 2 days for three times, mice were sacrificed prior to subject to tumourigenesis measurements. Tumour size (I), the percentage of TUNEL‐positive cells (J) or the percentage of Ki67‐positive cells (K) were assessed. Percentage of viable cells by flow cytometry represents the data normalised to vehicle Ctrl. All experiments were repeated three times, and graph with error bars show the data represent the mean ± standard deviation (SD) from technical triplicates (^*^ *p* < .05; ^**^ *p* < .01; ^***^ *p* < .001).

Despite increasing evidence demonstrating the diverse cellular roles of SOX9 in tumourigenesis and chemoresistance of various solid tumours, its role in DLBCL chemoresistance remains unclear. Therefore, we explored whether SOX9 contributes to DLBCL chemoresistance. t(14;18)‐positive Karpas‐422 (GCB) and DB cells (GCB) expressing high endogenous SOX9 were resistant to chemotherapeutic agents and BCL2 inhibitors. In contrast, t(14;18)‐negative OCI‐LY1 (GCB) and SUDHL6 (GCB) cells expressing low endogenous SOX9 were sensitive to Doxorubicin (DOX) and BCL2 inhibitors (Figure 2A‒C). To explore whether SOX9 regulates the drug sensitivity of DLBCL cells, lentiviral vectors encoding scrambled controls, shSOX9#1 or shSOX9#2, were transduced into Karpas‐422 or DB cells to stably knockdown SOX9. As shown in Figures 2D‒F and S2A, SOX9‐silenced Karpas‐422 and DB cells demonstrated higher sensitivity to chemotherapeutic agents (DOX and R‐CHOP) and BCL2 inhibitors than the scrambled control. In contrast, SOX9 overexpression in OCI‐LY1 cells resulted in resistance to chemotherapeutic agents (DOX and R‐CHOP) and BCL2 inhibitors (Figures 2G,H and S2B). To further validate that SOX9 inhibition increased the drug sensitivity of DLBCL cells in vivo, nude mice were transplanted with SOX9‐silenced DB cells. The mice were then administered the vehicle control or DOX. Interestingly, xenografts transplanted with SOX9‐silenced DLBCL were more sensitive to DOX and ABT‐199, displaying a smaller tumour size, reduced tumour cell proliferation and increased apoptosis (Figure 2I‒K). These data indicated that SOX9 contributed to the resistance of DLBCL cells with high BCL2 expression to standard chemotherapy and BCL2 inhibitors.

### 3.3 IRF4‐mediated BCL2‐induced SOX9 expression in DLBCL

**FIGURE 3:**
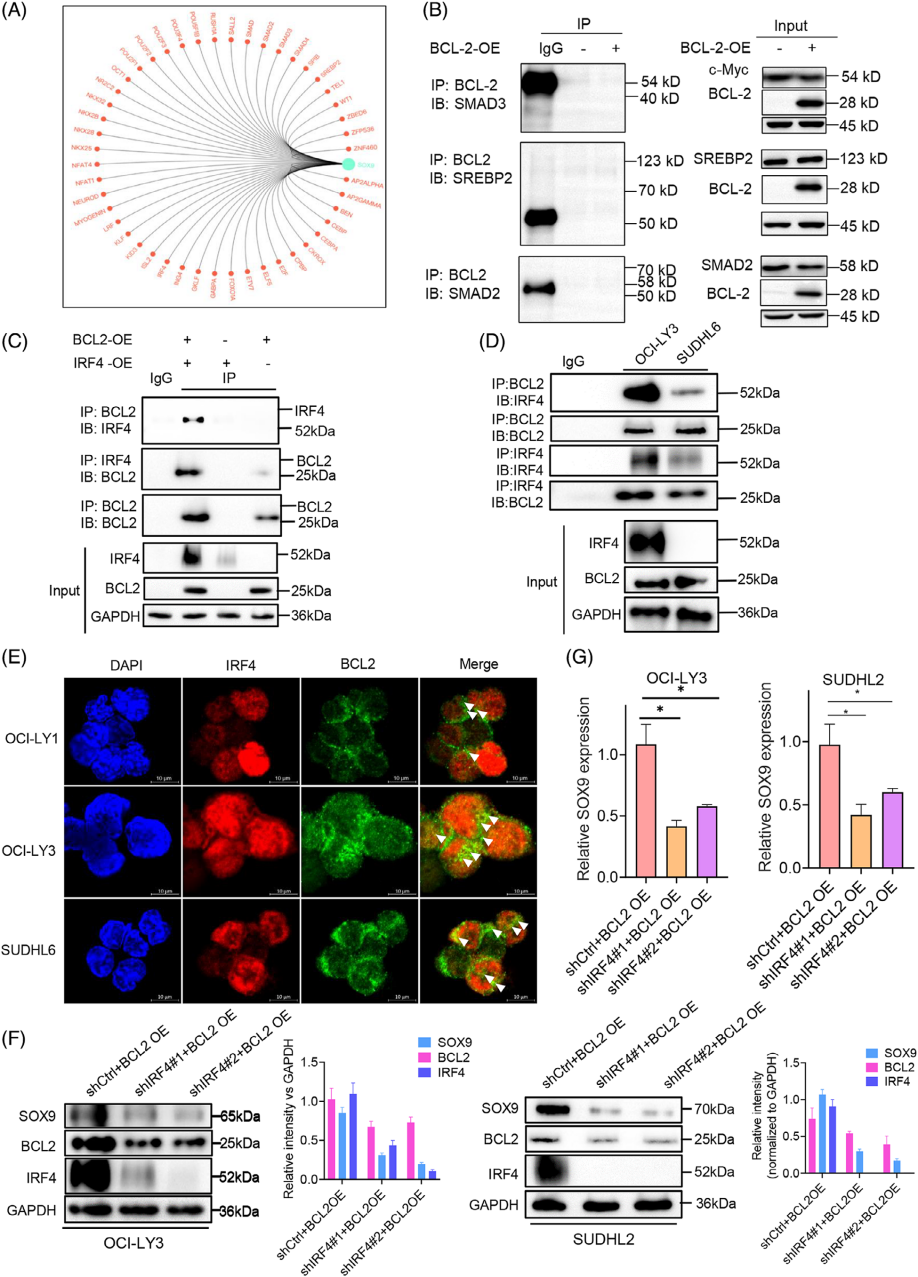
IRF4 mediates BCL2‐induced sex‐determining region Y (SRY)‐box 9 protein (SOX9) expression in diffuse large B‐cell lymphoma (DLBCL). (A) Bioinformatics representative schematic showing the potential upstream regulators of SOX9. (B) Co‐immunoprecipitation (co‐IP) assay was performed to identify the potential interacting partner of BCL2. (C) Whole cell lysates were extracted from 293T cells that either co‐transfected with or without PCDH‐BCL2, mCherry‐IRF4 lentiviral plasmids prior to immunoprecipitated either with anti‐BCL2, anti‐IRF4 or immunoglobulin G (IgG) isotype control antibodies overnight followed by immunoblotting assay. (D) Whole cell lysates were extracted from either OCI‐LY3 or SUDHL6 cells prior to immunoprecipitated either with anti‐BCL2, anti‐IRF4 or IgG isotype control antibodies overnight followed by immunoblotting assay. (E) Immunofluorescence assay in a panel of DLBCL cell lines (OCI‐LY1, OCI‐LY3 and SUDHL6). Red: anti‐IF4 antibody; green: anti‐BCL2 antibody; blue: DAPI. Arrows indicate co‐localisation areas. OCI‐LY3 or SUDHL2 cells were co‐transduced either with lentiviral encoding shCtrl/BCL2 OE, shIRF4#1/BCL2 OE or shIRF4#2/BCL2 OE plasmids for 72 h, followed either by (F) immunoblotting or (G) real‐time polymerase chain reaction (PCR) assay to determine the protein levels of SOX9, BCL2 and IRF4. Two‐way analysis of variance (ANOVA) was performed to compare the difference between shCtrl/BCL OE to shIRF4#1/BCL2 OE and shIRF4#2/BCL2 OE. β‐Actin or GAPDH was included as indication of equal loading. Protein levels were quantified (normalised to housekeeping genes) using ImageJ software and graph was generated by GraphPad version 9.0. All experiments were repeated three times, and graph with error bars show the data represent the mean ± standard deviation (SD) from technical triplicates (^*^ *p* < .05).

BCL2 is a critical regulator that mediates the apoptotic signalling pathway in mitochondria. BCL2 can regulate transcription via interactions with certain transcription factors such as NF‐κB^18^ and E2F1.^19^ As BCL2 upregulates SOX9 transcription, the key transcription factor mediating BCL2‐induced SOX9 expression in DLBCL cells was screened. Bioinformatic analysis was performed to predict the potential upstream SOX9 regulators, among which SMAD3, SREBP2, SMAD2 and IRF4 were selected as the top‐ranking factors for further validation (Figure 3A). Co‐immunoprecipitation assays were performed to determine which transcription factors were directly associated with BCL2. As demonstrated in Figure 3B, SMAD3, SREBP2 and SMAD2 showed no interactions with BCL2 (Figure 3B). BCL2 overexpression is not limited to *IGH::BCL2* rearrangement but also results from BCL2 gene amplification or mutations in the open reading frame that affect BCL2 interactions with other proteins.^31^ To confirm the interaction between endogenous BCL2 and IRF4 proteins, a co‐immunoprecipitation assay was performed using DLBCL cells expressing both BCL2 and IRF4. As shown in Figure 3C,D, both exogenous and endogenous IRF4 bands were observed upon BCL2 enrichment, suggesting that IRF4 interacts with BCL2 in DLBCL and may play a regulatory role in the induction of SOX9 expression by BCL2. To explore the subcellular compartments where they interact with each other, we performed immunofluorescence assays in OCI‐LY1, OCI‐LY3 and SUDHL6 cells, which express BCL2 and IRF4 proteins (Figure S3). As illustrated in Figure 3E, although IRF4 was predominantly localised in the cell nucleus, BCL2 was predominantly expressed in the cytoplasm and the two proteins co‐localised in the nucleus, represented by a yellowish colour. To further validate the essential role of IRF4 in BCL2‐induced SOX9 expression in DLBCL, we co‐transduced t(14;18)‐negative OCI‐LY3 (ABC) and SUDHL2 cells (ABC), two cells lines that exhibit high endogenous IRF4 level, with BCL2‐OE/shCtrl, BCL2‐OE/shIRF4#1 or BCL2‐OE/shIRF4#2 for 72 h. Silencing IRF4 attenuated BCL2‐induced SOX9 protein levels in DLBCL cells compared to the scrambled control (quantification images shown in Figure 3F). IRF4 suppression also counteracted BCL2‐induced SOX9 mRNA levels in DLBCL cells compared to those in scrambled control cells (Figure 3G). These data indicated that IRF4 may be a key BCL2 downstream transcription factor for SOX9 in the *IGH::BCL2*‐positive GCB DLBCL subtype.

### 3.4 IRF4‐regulated SOX9 transcription via binding to its promoter region

**FIGURE 4:**
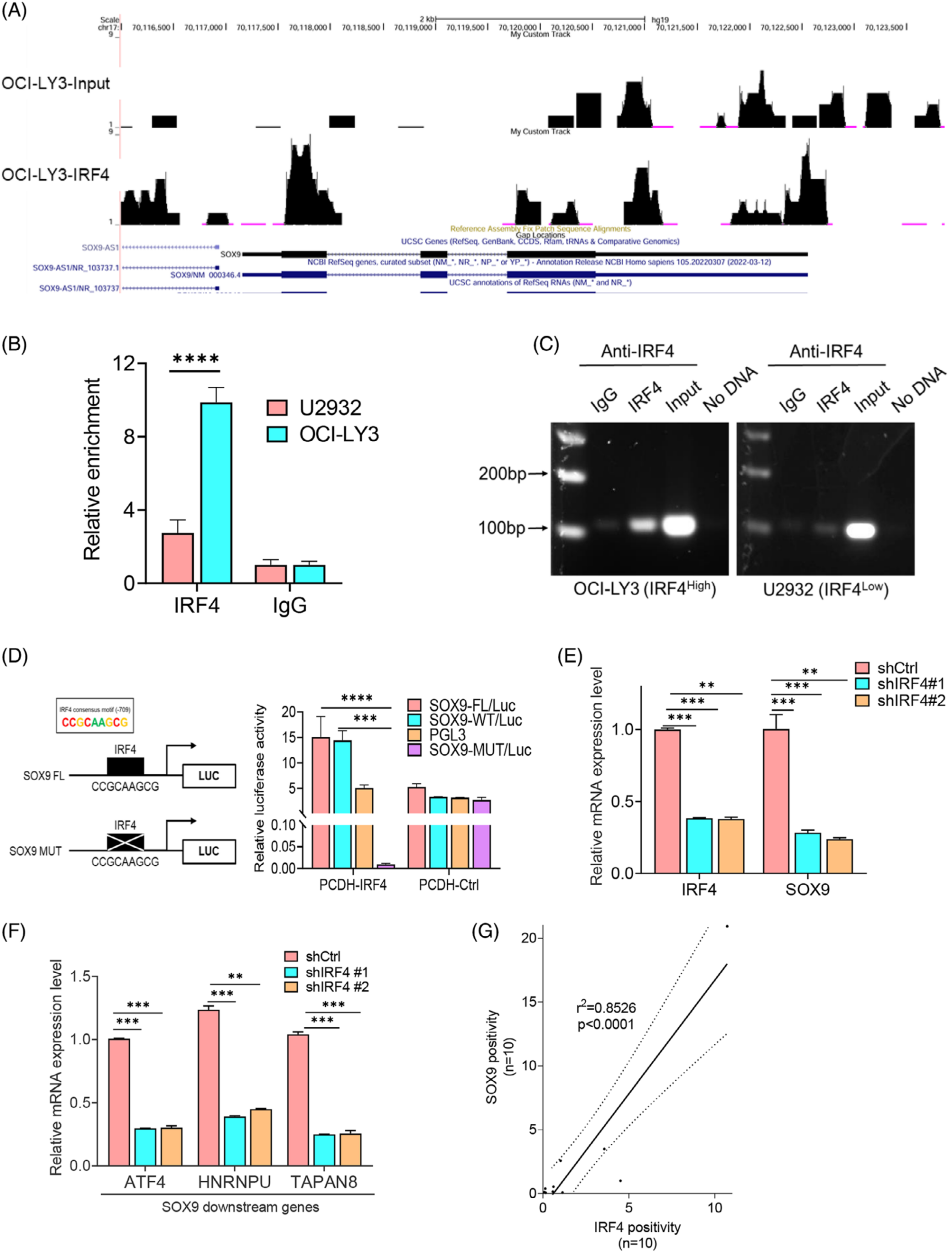
IRF4 regulates sex‐determining region Y (SRY)‐box 9 protein (SOX9) transcription via binding to its promoter region. (A) Binding profiles and peak calling records of IRF4 in the SOX9 promoter. (B and C) Chromatin immunoprecipitation (ChIP) assay validation of the IRF4 enrichment and binding site in the promoter of SOX9 in U2932 (IRF4 low expressing cells) and OCI‐LY3 (IRF4 high expressing cells). (D) Luciferase reporter plasmids carrying wild‐type (WT) SOX9 promoter or SOX9 promoter with mutated IRF4‐binding site was co‐transfected with pCDH‐IRF4, or vector control lentiviral plasmids in 293T cells. Luciferase activities were measured 48 h after transfection and normalised against firefly luciferase activities. Schematic representation of IRF4 consensus binding motif on the promoter of SOX9, SOX9 full length (FL) and SOX9 mutant luciferase reporter plasmids is shown. One‐way analysis of variance (ANOVA) was used to compare the difference between FL to MUT or WT to MUT. (E and F) OCI‐LY3 cells were transduced either with scramble control, shIRF4#1, shIRF4#2 or shIRF4#3 lentivirus plasmids for 72 h followed by real‐time polymerase chain reaction (PCR) of gene expressions of SOX9 and its target genes. (G) Immunohistochemical staining of IRF4 and SOX9 proteins in a cohort of de novo diffuse large B‐cell lymphoma (DLBCL) patients (*n* = 10), the positivity was measured by ImageJ and correlation graph was generated by GraphPad software 10.0. Two‐way ANOVA was used to compare the difference between shCtrl and shIRF4s. All experiments were repeated three times, and graph with error bars show data represent the mean ± standard deviation (SD) from technical triplicates (^**^ *p* < .01; ^***^ *p* < .001; ^****^ *p* < .005).

ChIP sequencing (ChIP‐seq) was performed on the t(14;18)‐negative OCI‐LY3 cells (ABC), which highly expresses endogenous IRF4 protein, to identify genes directly targeted by IRF4. Peak calling by model‐based analysis of the ChIP‐seq algorithm revealed 21 961 transcription sites that directly bound IRF4 (accession number GSE278092). The chr6:426522‐427104 and chr6:498262‐499280 SOX9 transcription start sites were highly enriched for IRF4 binding (Figure 4A). IRF4 binding to the SOX9 promoter was validated by ChIP using OCI‐LY3 cells. t(14;18)‐negative U2932 cells (ABC) exhibited a relatively low IRF4 level compared to OCI‐LY3 cells, which were included to clarify the specificity of IRF4‐binding activity. We identified a putative IRF4‐binding site using a previously published consensus IRF4‐binding motif (CCGCAAGCG). Amplification of the SOX9 promoter segment was observed in both DLBCL cell lines but with a much stronger intensity in OCI‐LY3 than in U2932 cells (Figure 4B,C). These results provided direct evidence that IRF4 binds to the SOX9 promoter. To further confirm that SOX9 is an IRF4 target, we constructed luciferase reporters containing the wild‐type SOX9 promoter encompassing a consensus‐binding motif of IRF4 or the SOX9 promoter harbouring a mutated (CCGCATCGC) IRF4 consensus motif (Figure 4D, left panel). Wild‐type or mutant IRF4 reporter plasmids were co‐transfected with pCDH‐IRF4 or empty vector lentiviral plasmids into 293T cells and luciferase activity measured at 48 h. IRF4 overexpression resulted in a significant increase in wild‐type SOX9 luciferase reporter activity (Figure 4D, right panel). In contrast, IRF4 overexpression failed to induce luciferase activity with the mutant SOX9 promoter (Figure 4D, right panel). Real‐time quantitative PCR demonstrated that inhibiting IRF4 downregulated SOX9, along with other well‐documented downstream target genes (Figure 4E,F). These results indicated that IRF4 is a direct transcription factor of SOX9.

### 3.5 BCL2‐promoted IRF4 nuclear translocation by enhancing its protein stability

**FIGURE 5:**
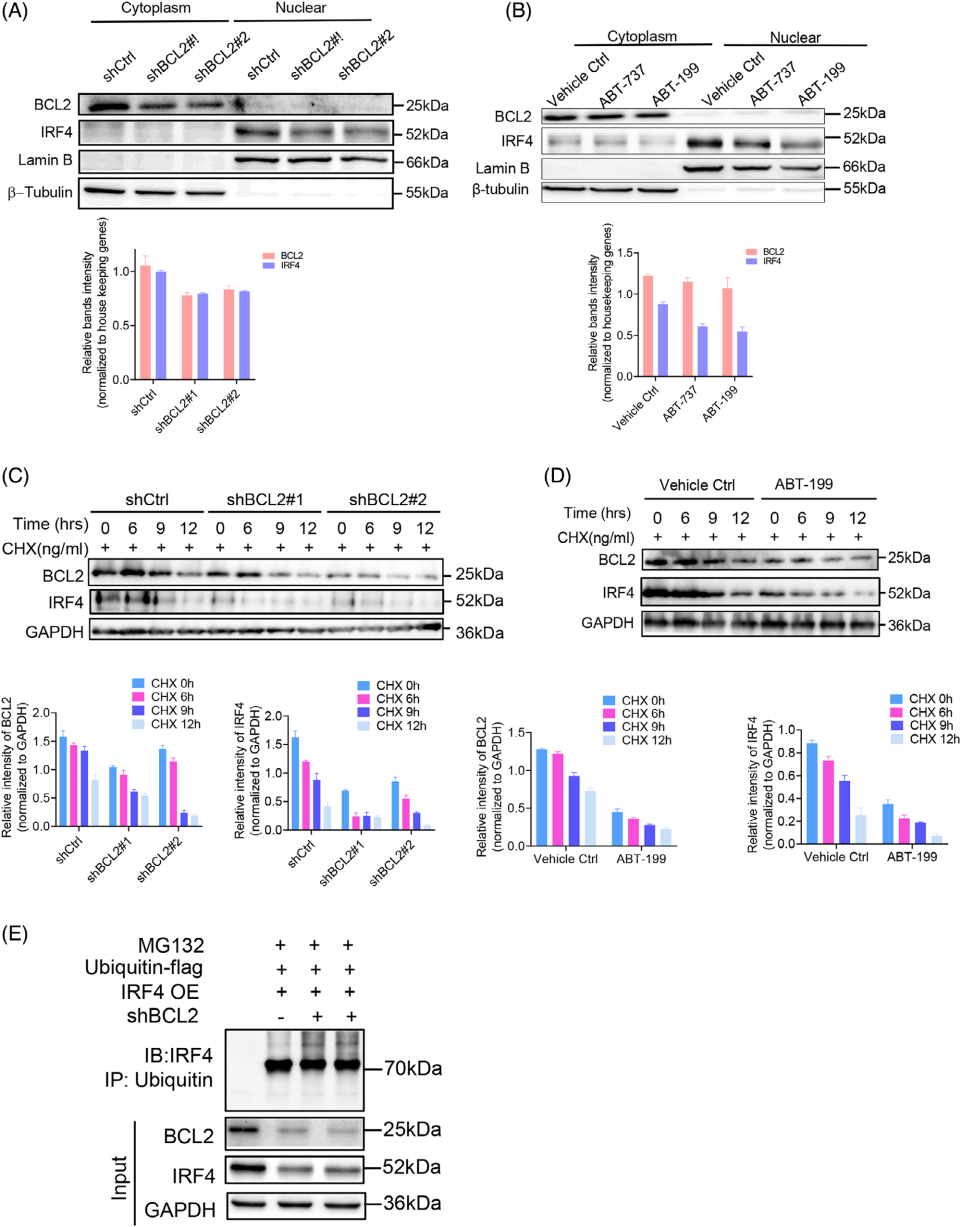
BCL2 promotes the nuclear translocation of IRF4 by enhancing its protein stability. OCI‐LY3 cells were transduced either with lentiviral encoding scramble control, shBCL2#1, shBCL2#2 plasmids for 72 h prior to subject to flow cytometry to sort out GFP‐positive cells to generate stable transfectants. OCI‐LY3 stable transfectants were either treated with vehicle control or ABT‐199 for 48 h, respectively, prior to subject to (A and B) immunoblotting using anti‐BCL2, anti‐IRF4 antibodies or (C and D) with addition of 300 ng/mL Cycloheximide (CHX) at the indicated time points followed by immunoblotting. Nuclear protein Lamin B or cytoplasmic protein β‐tubulin were also included as controls. (E) OCI‐LY3 cells were co‐transduced with lentiviral encoding flag‐ubiquitin, IRF4 or shBCL2 plasmids for 72 h followed by 20 mM of MG132 for additional 4 h. Cell lysates were harvested, and equal amounts of proteins were subjected to immunoprecipitation assay. A 10% input protein was loaded to determine the expressions of endogenous BCL2, IRF4. GAPDH was also included as an indication of equal loading. Protein levels (normalised to housekeeping genes) were quantified using ImageJ software and graph was generated using GraphPad version 9.0. All experiments were repeated three times, and graph with error bars show data represent the mean ± standard deviation (SD) from technical triplicates (^**^ *p* < .01; ^***^ *p* < .001; ^****^ *p* < .005) compared shCtrl to shIRF4s by two‐way analysis of variance (ANOVA).

To elucidate the consequences of the interaction between BCL2 and IRF4, we investigated IRF4 nuclear activity in BCL2‐silenced OCI‐LY3 cells, which endogenously express BCL2 and IRF4. Unsurprisingly, reduced endogenous BCL2 protein levels in the cytoplasmic fraction due to gene silencing attenuated IRF4 expression in the nuclear fraction compared to its scrambled control counterparts (Figure 5A). To confirm this finding, we treated OCI‐LY3 cells with 2.5 µM ABT‐737 or ABT‐199 for 48 h. Repressing BCL2 activity also reduced IRF4 protein levels in the nuclear extraction (Figure 5B). These data suggested that BCL2 enhanced IRF4 nuclear translocation in DLBCL cells. To explore the underlying molecular mechanism by which BCL2 promotes IRF4 nuclear translocation in DLBCL, we tested whether inhibiting BCL2 affected IRF4 protein stability. Silencing or inhibiting BCL2‐promoted endogenous IRF4 degradation (Figure 5C,D). To verify that BCL2 inhibits IRF4 degradation through the ubiquitin‒proteasome pathway, t(14;18)‐negative OCI‐LY3 cells (ABC) were co‐transduced with shBCL2#1/ubiquitin‐Flag, shBCL2#2/ubiquitin‐Flag or IRF4‐OE/ubiquitin‐Flag for 72 h, followed by MG132 treatment. BCL2 knockdown increased IRF4 polyubiquitination, providing evidence that BCL2 stabilises the IRF4 protein by inhibiting the ubiquitin‒proteasome pathway (Figure 5E).

### 3.6 SOX9 overexpression counteracted the IRF4‐silencing‐induced phenotype

**FIGURE 6:**
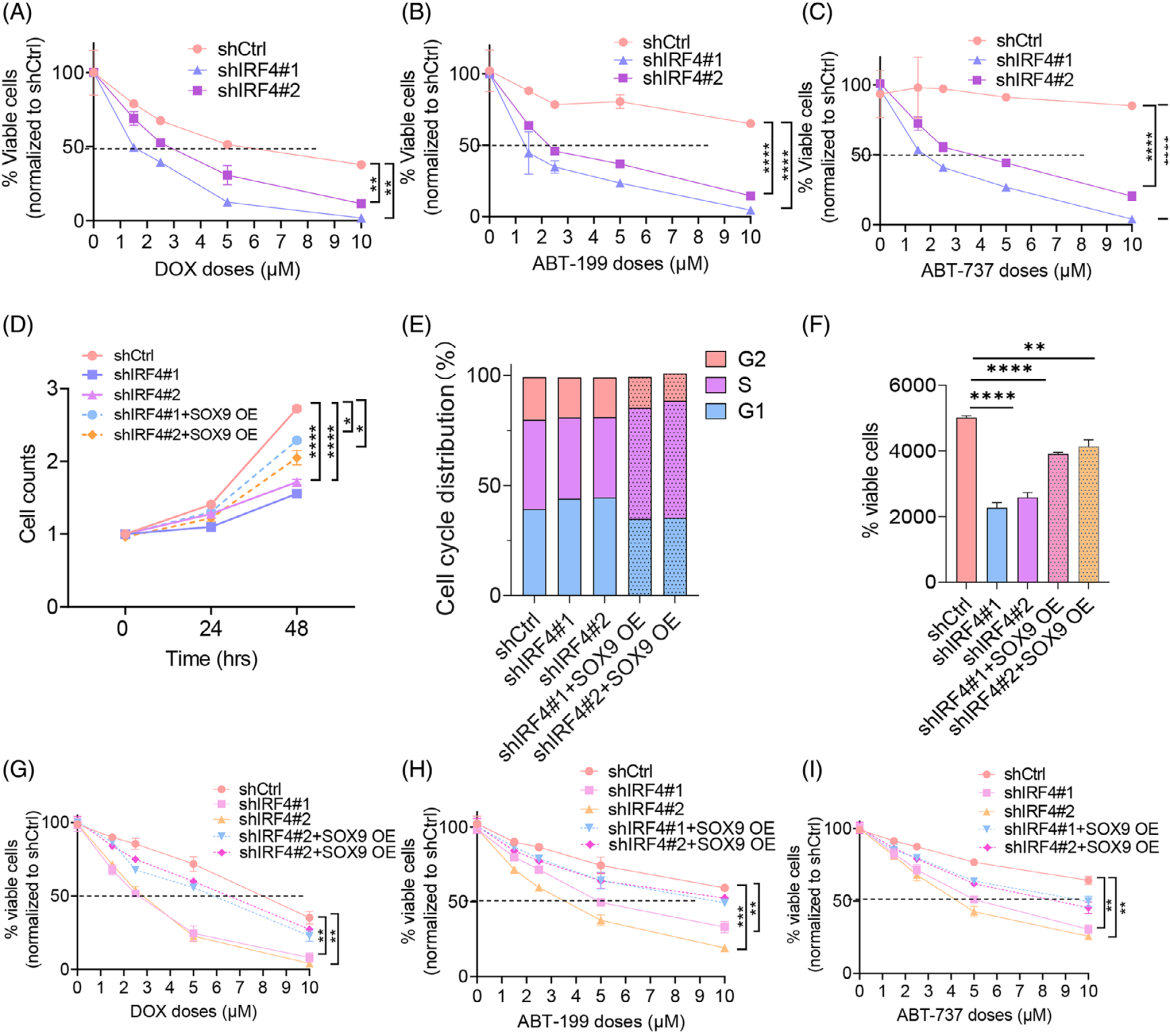
Sex‐determining region Y (SRY)‐box 9 protein (SOX9) overexpression counteracts IRF4‐silencing‐induced phenotype. OCI‐LY3 cells were transduced either with lentiviral encoding scramble control, shIRF4#1, shIRF4#2 or co‐transduced with shIRF4#1+SOX9 OE, or shIRF4#2+SOX9 for 72 h, respectively, prior to subject to (A‒C) drug sensitivity assays (D) cell growth, (E) cell cycle and (F) cell viability assays. (G‒I) Drug sensitivity assay by flow cytometry. All experiments were repeated three times, and graph with error bars show data represent the mean ± standard deviation (SD) from technical triplicates (^*^ *p* < .05; ^**^ *p* < .01; ^****^ *p* < .005) compare either scramble control to shIRF4#1 or shIRF4#2, or to shIRF4#1+SOX9 OE or shIRF4#2+SOX9 OE by two‐way analysis of variance (ANOVA).

To determine the effects of IRF4 on DLBCL chemoresistance, we established OCI‐LY3 stable transfectants with lentiviral vectors encoding scrambled controls shIRF4#1 and shIRF4#2, followed by treatment with chemotherapy (DOX and R‐CHOP) or BCL2 inhibitors for 48 h. Figures 6A‒C and S2C show that IRF4‐silenced OCI‐LY3 cells were more sensitive to chemotherapeutic agents and BCL2 inhibitors than their scrambled control counterparts. We investigated whether SOX9 plays a central role in counterbalancing the biological effects of silencing IRF4 in DLBCL. We transduced t(14;18)‐negative OCI‐LY3 cells (ABC) with shCtrl, shIRF4#1 or shIRF4#2 alone or co‐transduced OCI‐LY3 cells with shIRF4#1/SOX9 OE or shIRF4#2/SOX9 OE for 72 h before functional analysis. SOX9 rescued IRF4 knockdown‐induced DLBCL cell growth inhibition, G_1_/S cell cycle arrest and cell apoptosis (Figure 6D‒F). SOX9 overexpression impaired IRF4 knockdown‐induced chemosensitivity (Figures 6G‒I and S2D). These findings provide strong evidence of the pivotal role of SOX9 in mediating IRF4 function in DLBCL.

### 3.7 Targeting IRF4‐impeded lymphomagenesis and resensitised DLBCL to chemotherapy

**FIGURE 7:**
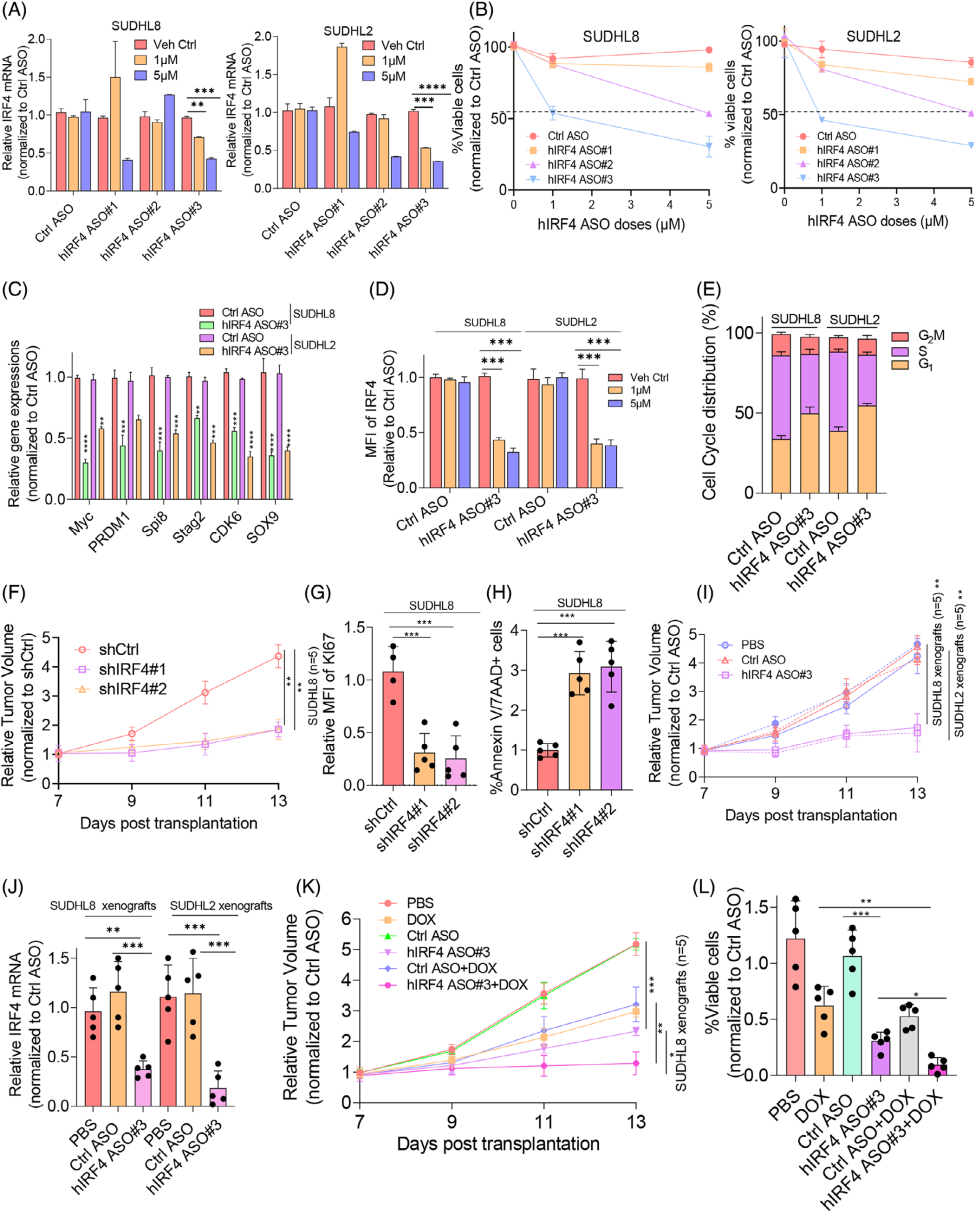
In vivo targeting IRF4 impedes lymphomagenesis and resensitising diffuse large B‐cell lymphoma (DLBCL) to chemotherapy. SUDHL8 and SUDHL2 cells were incubated either with vehicle control, 1 or 5 µM Ctrl ASO, hIRF4 ASO#1, hIRF4 ASO#2 or hIRF4 ASO#3 for 72 h, respectively, followed by (A) real‐time polymerase chain reaction (PCR) analysis of IRF4 mRNA level, (B) flow cytometry assay to determine cell viability, (C) expressions of IRF4 target genes, (D) MFI of IRF4 or (E) cell cycle distribution. Ten million of IRF4 deficient OCI‐LY3 cells were subcutaneously injected into each nude mouse (*n* = 5). Three weeks post‐transplantation, mice were sacrificed and (F) tumour volume, (G) tumour cell proliferation and (H) apoptosis were measured. SUDHL8 (solid lines) and SUDHL2 (dash lines) DLBCL xenografts were administrated either with Ctrl ASO (25 mg/kg) or hIRF4 ASO (25 mg/kg) five times per week for 3 weeks, mice were sacrificed prior to tumourigenesis indexes measurement as demonstrated in (I) tumour volume and (J) IRF4 mRNA level. SUDHL8 (solid lines) and SUDHL2 (dash lines) DLBCL xenografts were administrated either with Ctrl ASO (25 mg/kg) or hIRF4 ASO (25 mg/kg) five times per week for 3 weeks or DOX (2.5 mg/kg) every 2 days for four times alone, or combination. Phosphate‐buffered saline (PBS) control was also included as a vehicle control. Three weeks post‐drug administration, mice were sacrificed prior to subject to tumourigenesis indexes measurements. (K) Tumour volume and (L) cell viability was assessed. All experiments were repeated three times, and graph with error bars show data represent the mean ± standard deviation (SD) from technical triplicates (^*^ *p* < .01; ^**^ *p* < .05; ^***^ *p* < .001; ^****^ *p* < .005).

To selectively inhibit IRF4 in DLBCL as a therapeutic strategy, three human IRF4‐specific ASOs (hIRF4‐ASO#1, hIRF4‐ASO#2 and hIRF4‐ASO#3) were tested for their therapeutic activity in vitro and in vivo. Two DLBCL cell lines exhibiting relatively high endogenous IRF4 protein levels were treated with IRF4‐ASOs for 72–96 h. hIRF4‐ASO#3 displayed the most significant decrease in IRF4 mRNA and protein expression, concomitant with reduced cell viability, cell cycle progression and IRF4‐targeted gene expression in a dose‐dependent manner (Figure 7A‒E). To explore the biological significance of IRF4 inhibition in vivo, shIRF4 lentivirus‐transduced SUDHL8 cells were subcutaneously injected into nude mice (*n* = 5). Three weeks post‐transplantation, the DLBCL xenograft mice were killed and tumourigenesis‐related phenotypes measured. Tumour volume was dramatically reduced in shIRF4‐transduced SUDHL8‐xenografted mice compared to that in controls (Figure 7F). Increased cell death and decreased proliferation of DLBCL cells were observed in IRF4‐deficient OCI‐LY3‐xenografted mice (Figure 7G,H). To validate the therapeutic effect of IRF4‐targeted ASOs in vivo, SUDHL8 or SUDHL2 cells were transplanted into nude mice (*n* = 5), as described previously.^29^ Three weeks post‐transplantation, the xenografted mice were randomised and administered hIRF4‐ASOs. Impressively, hIRF4‐ASOs significantly reduced tumour volume and lowered IRF4 mRNA levels (Figure 7I,J). To elucidate whether hIRF4‐ASOs synergised with DOX, we treated SUDHL8 xenografts with DOX and hIRF4‐ASOs alone or in combination. Figure 7K,L shows that hIRF4‐ASOs synergised with DOX and significantly affected tumour volume and cell survival compared with the monotherapy regimen of DOX or hIRF4‐ASO. In summary, our in vivo data demonstrated that targeting IRF4 may be a promising strategy to overcome chemoresistance in DLBCL cells.

## 4 DISCUSSION

Our previous study in a large DLBCL cohort revealed that SOX9 was highly expressed in BCL2‐overexpressing DLBCL subsets, providing evidence that BCL2 may modulate SOX9 expression. Herein, we demonstrated that SOX9 enhanced drug resistance in BCL2‐overexpressing DLBCL via a novel transcription factor, IRF4, by mediating BCL2‐induced SOX9 transcription. BCL2 promotes IRF4 entry into the nucleus by enhancing its protein stability and downregulating the proteasome ubiquitination process, thus enforcing SOX9‐induced phenotypes. Consequently, pharmacological targeting of IRF4 using hIRF4‐ASOs impaired lymphomagenesis and resensitised DLBCL cells to chemotherapy. These findings imply the importance of IRF4 as a novel and promising therapeutic target for treating BCL2‐overexpressing DLBCL with a worse prognosis.

Accumulating evidence indicates a correlation between BCL2 and SOX9 in various disease models, such as chondrocyte and bone diseases.^32^, ^33^, ^34^ However, the relationship between BCL2 and SOX9 in haematological malignancies, especially B‐cell lymphoma, is poorly understood. Our previous study on SOX9 expression in a large number of DLBCL patient samples revealed high SOX9 expression in a subset of patients with GCB DLBCL harbouring the IGH/BCL2 translocation, which frequently results in elevated BCL2 expression. We showed that inhibiting BCL2 downregulated SOX9 protein and mRNA expression in BCL2‐overexpressing DLBCL, indicating that SOX9 induction was significantly associated with BCL2 rearrangement in the GCB DLBCL subset. However, the variation in BCL2 positivity was potentially due to the B‐cell lymphoma heterogeneity or the BCL2 mutation altering the immunohistochemical binding site epitope of the antibody. Studies involving large cohorts and antibodies with different epitopes should be used to validate these findings.^35^

A substantial amount of data has revealed that SOX9 contributes to chemoresistance in various solid tumour models, including gastric cancer,^23^, ^36^ hepatocellular carcinoma,^37^ pancreatic cancer^38^ and glioma.^39^ Tumour cells bearing the *IGH::BCL2* translocation are more dependent on BCL2 for survival and are more sensitive to BCL2 inhibitors.^40^ We demonstrated that SOX9 enhanced resistance of the BCL2‐overexpressing DLBCL subset against standard chemotherapy as well as a BCL2 inhibitor, indicating that DLBCL cells expressing high SOX9 levels rely on BCL2 to survive and further clarifying its oncogenic role in lymphomagenesis.

Although we previously found that SOX9 is preferentially overexpressed in DLBCL with *IGH::BCL2* translocation,^29^ the underlying molecular mechanisms remain unclear. Our study identified IRF4 as a novel transcription factor responsible for SOX9 induction in BCL2‐overexpressing DLBCL. Despite reports showing that IRF4 plays an oncogenic role in promoting lymphomagenesis via downregulation of BCL2 family proteins, such as BIM and BMF,^41^ we revealed that IRF4 directly interacts with BCL2 in DLBCL. More importantly, BCL2 promotes IRF4 nuclear translocation by enhancing its protein stability. These data demonstrated that IRF4 is a novel BCL2 downstream regulator that mediates SOX9‐induced drug resistance.

Increasing amounts of data suggest an oncogenic role for IRF4 in B‐cell malignancies.^42^, ^43^, ^44^ However, the molecular mechanisms by which IRF4 drives lymphomagenesis remain unclear. Our results indicated that SOX9 is a novel IRF4 regulatory target gene by showing that IRF4 directly controls SOX9 transcription by binding to the SOX9 promoter, which consequently activates SOX9‐induced lymphomagenesis.

Our findings demonstrated that IRF4‐mediated SOX9 induction in BCL2‐overexpressing DLBCL was associated with tumour progression, suggesting the potential therapeutic implication of the IRF4‒SOX9 signalling axis in this type of DLBCL. Because transcription factors are extremely difficult to target with small inhibitors, we prevented IRF4 transcription with an IRF4‐selective ASO that could be directly delivered into cells through free uptake and has shown efficacy in cancers such as multiple myeloma.^45^ We showed that IRF4‐ASOs significantly reduced tumour volume and downregulated IRF4 transcription in xenograft models. To overcome high‐dose chemotherapy‐induced toxicity, we combined IRF4‐ASOs with DOX, which resulted in better antitumour activity than single‐drug therapy. These findings indicate that IRF4 can be exploited as a promising therapeutic target to ameliorate the antitumour efficacy of currently used chemotherapeutic agents. Despite the relatively low positivity of IRF4 in BCL2 overexpressed cases (from 18% to 44%),^46^, ^47^ as BCL2 overexpression is not only found in DLBCL harbouring *IGH::BCL2* translocation but also resulted from BCL2 gene amplification or ORF mutation,^31^ IRF4‐targeted therapy may be beneficial in these DLBCL subsets.

In conclusion, our in‐depth study revealed that BCL2 activated IRF4 by enhancing its nuclear activity to induce SOX9 aberrant expression, which is a critical pathway for drug resistance in BCL2‐overexpressing DLBCL. Targeting IRF4 may be worth investigating further regarding its potential to overcome the chemoresistance of BCL2‐overexpressing DLBCL to standard therapies (see Graphical Abstract).

## AUTHOR CONTRIBUTIONS

Yirong Zhang and Zizhen Xu performed experiments and analysed the data. Ruixin Sun and Yixuan Gao performed experiments and participated partially in data analysis. Agida Innocent and Kasimujiang Aximujiang proofread the manuscript and contributed valuable thoughts on the project design. Lin Yuan conceptualised the project, collected additional clinical samples, applied ethical approval, accumulated patients’ clinical information and wrote part of the manuscript. Jiao Ma conceptualised, directed the project, designed experiments and wrote manuscript.

## CONFLICT OF INTEREST STATEMENT

The authors declare they have no conflicts of interest.

## ETHICS STATEMENT

This study was conducted in accordance with the Declaration of Helsinki regulations of the protocols approved by Shanghai General Hospital after Institutional Review Board review and approval (2024‐355). Patients’ clinical Information was obtained from electronic clinical records, and written consent for use of the samples for research was obtained from patients or their guardians. All mice experiments were performed under an Institutional Animal Care and Use Committee‐approved protocol, and institutional guidelines for the proper use of animals in research were followed (JUMC2023‐127‐A).

## Supporting information



Supporting Information

Supporting Information

Supporting Information

Supporting Information

Supporting Information

## Data Availability

ChIP‐seq data were deposited in GEO database (accession number GSE278092).

## References

[1] Usuda D , Izumida T , Terada N , et al. Diffuse large B cell lymphoma originating from the maxillary sinus with skin metastases: a case report and review of literature. World J Clin Cases. 2021;9(23):6886‐6899. 34447839 10.12998/wjcc.v9.i23.6886 PMC8362537

[2] Coiffier B , Lepage E , Briere J , et al. CHOP chemotherapy plus rituximab compared with CHOP alone in elderly patients with diffuse large‐B‐cell lymphoma. N Engl J Med. 2002;346(4):235‐242. 11807147 10.1056/NEJMoa011795

[3] Vodicka P , Klener P , Trneny M . Diffuse large B‐cell lymphoma (DLBCL): early patient management and emerging treatment options. Onco Targets Ther. 2022;15:1481‐1501. 36510607 10.2147/OTT.S326632 PMC9739046

[4] Zhang J , Gu Y , Chen B . Drug‐resistance mechanism and new targeted drugs and treatments of relapse and refractory DLBCL. Cancer Manag Res. 2023;15:245‐255. 36873252 10.2147/CMAR.S400013 PMC9976586

[5] Jakobsen LH , Ovlisen AK , Severinsen MT , et al. Patients in complete remission after R‐CHOP(‐like) therapy for diffuse large B‐cell lymphoma have limited excess use of health care services in Denmark. Blood Cancer J. 2022;12(1):16. 35087026 10.1038/s41408-022-00614-8 PMC8795387

[6] Atesoglu EB , Gulbas Z , Uzay A , et al. Glofitamab in relapsed/refractory diffuse large B‐cell lymphoma: real‐world data. Hematol Oncol. 2023;41(4):663‐673. 37211991 10.1002/hon.3174

[7] Schmitz R , Wright GW , Huang DW , et al. Genetics and pathogenesis of diffuse large B‐cell lymphoma. N Engl J Med. 2018;378(15):1396‐1407. 29641966 10.1056/NEJMoa1801445 PMC6010183

[8] Otto C , Scholtysik R , Schmitz R , et al. Novel IGH and MYC translocation partners in diffuse large B‐cell lymphomas. Genes Chromosomes Cancer. 2016;55(12):932‐943. 27356265 10.1002/gcc.22391

[9] Knittel G , Liedgens P , Korovkina D , Pallasch CP , Reinhardt HC . Rewired NFkappaB signaling as a potentially actionable feature of activated B‐cell‐like diffuse large B‐cell lymphoma. Eur J Haematol. 2016;97(6):499‐510. 27526684 10.1111/ejh.12792

[10] Lohr JG , Stojanov P , Lawrence MS , et al. Discovery and prioritization of somatic mutations in diffuse large B‐cell lymphoma (DLBCL) by whole‐exome sequencing. Proc Natl Acad Sci U S A. 2012;109(10):3879‐3884. 22343534 10.1073/pnas.1121343109 PMC3309757

[11] Laddaga FE , Ingravallo G , Mestice A , et al. Correlation between circulating blood and microenvironment T lymphocytes in diffuse large B‐cell lymphomas. J Clin Pathol. 2022;75(7):493‐497. 34011621 10.1136/jclinpath-2020-207048

[12] Scherer F , Kurtz DM , Newman AM , et al. Distinct biological subtypes and patterns of genome evolution in lymphoma revealed by circulating tumor DNA. Sci Transl Med. 2016;8(364):364ra155. 10.1126/scitranslmed.aai8545 PMC5490494 27831904

[13] Tsujimoto Y , Finger LR , Yunis J , Nowell PC , Croce CM . Cloning of the chromosome breakpoint of neoplastic B cells with the t(14;18) chromosome translocation. Science. 1984;226(4678):1097‐1099. 6093263 10.1126/science.6093263

[14] Pasqualucci L , Khiabanian H , Fangazio M , et al. Genetics of follicular lymphoma transformation. Cell Rep. 2014;6(1):130‐140. 24388756 10.1016/j.celrep.2013.12.027 PMC4100800

[15] Li S , Seegmiller AC , Lin P et al. B‐cell lymphomas with concurrent MYC and BCL2 abnormalities other than translocations behave similarly to MYC/BCL2 double‐hit lymphomas. Mod Pathol. 2015;28(2):208‐217. 25103070 10.1038/modpathol.2014.95

[16] Roh J , Cho H , Pak HK , et al. BCL2 super‐expressor diffuse large B‐cell lymphoma: a distinct subgroup associated with poor prognosis. Mod Pathol. 2022;35(4):480‐488. 34764434 10.1038/s41379-021-00962-z

[17] Braun F , de Carne Trecesson S , Bertin‐Ciftci J , Juin P . Protect and serve: bcl‐2 proteins as guardians and rulers of cancer cell survival. Cell Cycle. 2013;12(18):2937‐2947. 23974114 10.4161/cc.25972 PMC3875667

[18] Lee HH , Dadgostar H , Cheng Q , Shu J , Cheng G . NF‐kappaB‐mediated up‐regulation of Bcl‐x and Bfl‐1/A1 is required for CD40 survival signaling in B lymphocytes. Proc Natl Acad Sci U S A. 1999;96(16):9136‐9141. 10430908 10.1073/pnas.96.16.9136 PMC17745

[19] Youn CK , Cho HJ , Kim SH , et al. Bcl‐2 expression suppresses mismatch repair activity through inhibition of E2F transcriptional activity. Nat Cell Biol. 2005;7(2):137‐147. 15619620 10.1038/ncb1215

[20] Chen J , Xia P , Liu Y , Kogan C , Cheng Z . Loss of Rbl2 (retinoblastoma‐like 2) exacerbates myocardial ischemia/reperfusion injury. J Am Heart Assoc. 2022;11(19):e024764. 36129061 10.1161/JAHA.121.024764 PMC9673695

[21] Jo A , Denduluri S , Zhang B , et al. The versatile functions of Sox9 in development, stem cells, and human diseases. Genes Dis. 2014;1(2):149‐161. 25685828 10.1016/j.gendis.2014.09.004 PMC4326072

[22] Cui J , Christin JR , Reisz JA , et al. Targeting ABCA12‐controlled ceramide homeostasis inhibits breast cancer stem cell function and chemoresistance. Sci Adv. 2023;9(48):eadh1891. 38039374 10.1126/sciadv.adh1891 PMC10691781

[23] Feng Q , Cui N , Li S , Cao J , Chen Q , Wang H . Upregulation of SOX9 promotes the self‐renewal and tumorigenicity of cervical cancer through activating the Wnt/beta‐catenin signaling pathway. FASEB J. 2023;37(10):e23174. 37668416 10.1096/fj.202201596RRR

[24] Avendano‐Felix M , Aguilar‐Medina M , Romero‐Quintana JG , et al. SOX9 knockout decreases stemness properties in colorectal cancer cells. J Gastrointest Oncol. 2023;14(4):1735‐1745. 37720443 10.21037/jgo-22-1163 PMC10502562

[25] Zhong H , Lu W , Tang Y , et al. SOX9 drives KRAS‐induced lung adenocarcinoma progression and suppresses anti‐tumor immunity. Oncogene. 2023;42(27):2183‐2194. 37258742 10.1038/s41388-023-02715-5 PMC11809655

[26] Lu J , Liang T , Li P , Yin Q . Regulatory effects of IRF4 on immune cells in the tumor microenvironment. Front Immunol. 2023;14:1086803. 36814912 10.3389/fimmu.2023.1086803 PMC9939821

[27] Perini T , Materozzi M , Milan E . The immunity‐malignancy equilibrium in multiple myeloma: lessons from oncogenic events in plasma cells. FEBS J. 2022;289(15):4383‐4397. 34117720 10.1111/febs.16068

[28] Zhang Y , Shen Y , Wei W , et al. Dysregulation of SIRT3 SUMOylation confers AML chemoresistance via controlling HES1‐dependent fatty acid oxidation. Int J Mol Sci. 2022;23(15):8282. 35955415 10.3390/ijms23158282 PMC9368767

[29] Shen Y , Zhou J , Nie K , et al. Oncogenic role of the SOX9‐DHCR24‐cholesterol biosynthesis axis in IGH‐BCL2+ diffuse large B‐cell lymphomas. Blood. 2022;139(1):73‐86. 34624089 10.1182/blood.2021012327 PMC8740888

[30] Souers AJ , Leverson JD , Boghaert ER , et al. ABT‐199, a potent and selective BCL‐2 inhibitor, achieves antitumor activity while sparing platelets. Nat Med. 2013;19(2):202‐208. 23291630 10.1038/nm.3048

[31] Monni O , Franssila K , Joensuu H , Knuutila S . BCL2 overexpression in diffuse large B‐cell lymphoma. Leuk Lymphoma. 1999;34(1‐2):45‐52. 10350331 10.3109/10428199909083379

[32] Tare RS , Townsend PA , Packham GK , Inglis S , Oreffo RO . Bcl‐2‐associated athanogene‐1 (BAG‐1): a transcriptional regulator mediating chondrocyte survival and differentiation during endochondral ossification. Bone. 2008;42(1):113‐128. 17950682 10.1016/j.bone.2007.08.032

[33] Pan T , Chen R , Wu D et al. Alpha‐Mangostin suppresses interleukin‐1beta‐induced apoptosis in rat chondrocytes by inhibiting the NF‐kappaB signaling pathway and delays the progression of osteoarthritis in a rat model. Int Immunopharmacol. 2017;52:156‐162. 28915439 10.1016/j.intimp.2017.08.021

[34] Chang JK , Chang LH , Hung SH , et al. Parathyroid hormone 1–34 inhibits terminal differentiation of human articular chondrocytes and osteoarthritis progression in rats. Arthritis Rheum. 2009;60(10):3049‐3060. 19790062 10.1002/art.24843

[35] Maeshima AM , Taniguchi H , Furukawa H , et al. Diagnostic clues of BCL2‐negative, faint, or controversial follicular lymphomas: a study of 103 cases. Hum Pathol. 2023;135:84‐92. 36702355 10.1016/j.humpath.2023.01.006

[36] Fan Y , Li Y , Yao X , et al. Epithelial SOX9 drives progression and metastases of gastric adenocarcinoma by promoting immunosuppressive tumour microenvironment. Gut. 2023;72(4):624‐637. 36002248 10.1136/gutjnl-2021-326581

[37] Yan J , Xie B , Tian Y , et al. MicroRNA‐5195‐3p mediated malignant biological behaviour of insulin‐resistant liver cancer cells via SOX9 and TPM4. BMC Cancer. 2023;23(1):557. 37328795 10.1186/s12885-023-11068-x PMC10273698

[38] Carrasco‐Garcia E , Lopez L , Moncho‐Amor V , et al. SOX9 triggers different epithelial to mesenchymal transition states to promote pancreatic cancer progression. Cancers. 2022;14(4):916. 10.3390/cancers14040916 PMC8870732 35205666

[39] Li B , Zhao H , Song J , Wang F , Chen M . LINC00174 down‐regulation decreases chemoresistance to temozolomide in human glioma cells by regulating miR‐138‐5p/SOX9 axis. Hum Cell. 2020;33(1):159‐174. 31713817 10.1007/s13577-019-00281-1

[40] Huang J , Fairbrother W , Reed JC . Therapeutic targeting of Bcl‐2 family for treatment of B‐cell malignancies. Expert Rev Hematol. 2015;8(3):283‐297. 25912824 10.1586/17474086.2015.1026321

[41] Fedele PL , Liao Y , Gong JN , et al. The transcription factor IRF4 represses proapoptotic BMF and BIM to licence multiple myeloma survival. Leukemia. 2021;35(7):2114‐2118. 33149265 10.1038/s41375-020-01078-0

[42] Lossos IS . The endless complexity of lymphocyte differentiation and lymphomagenesis: IRF‐4 downregulates BCL6 expression. Cancer Cell. 2007;12(3):189‐191. 17785200 10.1016/j.ccr.2007.08.012

[43] Saito M , Gao J , Basso K , et al. A signaling pathway mediating downregulation of BCL6 in germinal center B cells is blocked by BCL6 gene alterations in B cell lymphoma. Cancer Cell. 2007;12(3):280‐292. 17785208 10.1016/j.ccr.2007.08.011

[44] Ricker E , Verma A , Marullo R , et al. Selective dysregulation of ROCK2 activity promotes aberrant transcriptional networks in ABC diffuse large B‐cell lymphoma. Sci Rep. 2020;10(1):13094. 32753663 10.1038/s41598-020-69884-1 PMC7403583

[45] Mondala PK , Vora AA , Zhou T , et al. Selective antisense oligonucleotide inhibition of human IRF4 prevents malignant myeloma regeneration via cell cycle disruption. Cell Stem Cell. 2021;28(4):623‐636.e9. 33476575 10.1016/j.stem.2020.12.017 PMC8026723

[46] Frauenfeld L , Castrejon‐de‐Anta N , Ramis‐Zaldivar JE , et al. Diffuse large B‐cell lymphomas in adults with aberrant coexpression of CD10, BCL6, and MUM1 are enriched in IRF4 rearrangements. Blood Adv. 2022;6(7):2361‐2372. 34654055 10.1182/bloodadvances.2021006034 PMC9006278

[47] Berg HE , Peterson JF , Lee HE , McPhail ED . Large B‐cell lymphoma with IRF4 gene rearrangements: differences in clinicopathologic, immunophenotypic and cytogenetic features between pediatric and adult patients. Hum Pathol. 2023;131:108‐115. 36470475 10.1016/j.humpath.2022.10.011

