# Supplementary material for: IRF4 contributes to chemoresistance in IGH::BCL2‐positive diffuse large B‐cell lymphomas by mediating BCL2‐induced SOX9 expression
Source: Clin Transl Med. 2025 May 12;15(5):e70336. doi: 10.1002/ctm2.70336 (PMC12069798; doi:10.1002/ctm2.70336)
Supplement: Supplementary file 1 — Supporting Information [file CTM2-15-e70336-s005.pptx]

## Slide 1
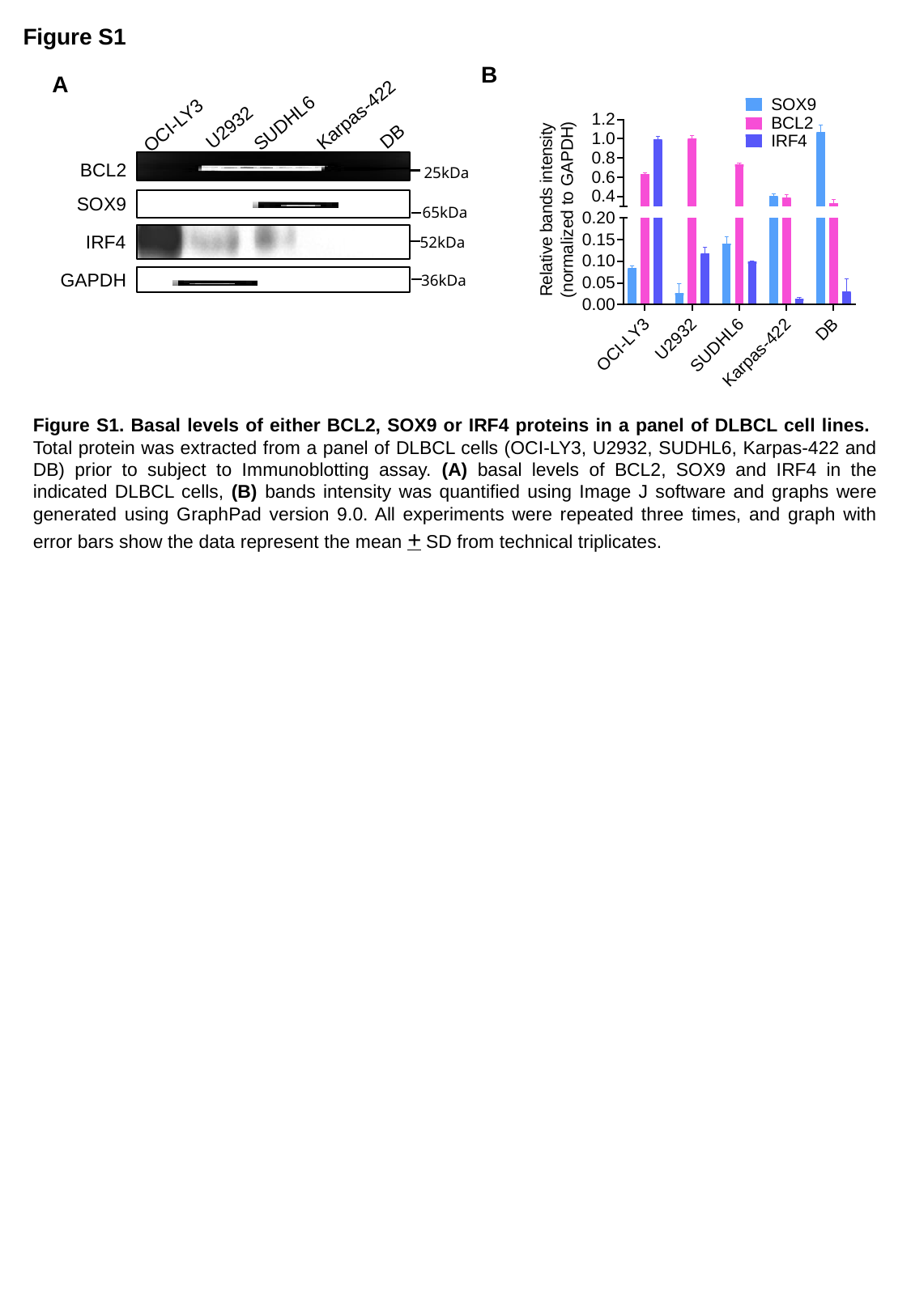

Figure S1
B
A
Karpas-422
SUDHL6
OCI-LY3
U2932
DB
BCL2
25kDa
SOX9
65kDa
IRF4
52kDa
GAPDH
36kDa
Figure S1. Basal levels of either BCL2, SOX9 or IRF4 proteins in a panel of DLBCL cell lines. Total protein was extracted from a panel of DLBCL cells (OCI-LY3, U2932, SUDHL6, Karpas-422 and DB) prior to subject to Immunoblotting assay. (A) basal levels of BCL2, SOX9 and IRF4 in the indicated DLBCL cells, (B) bands intensity was quantified using Image J software and graphs were generated using GraphPad version 9.0. All experiments were repeated three times, and graph with error bars show the data represent the mean + SD from technical triplicates.

## Slide 2
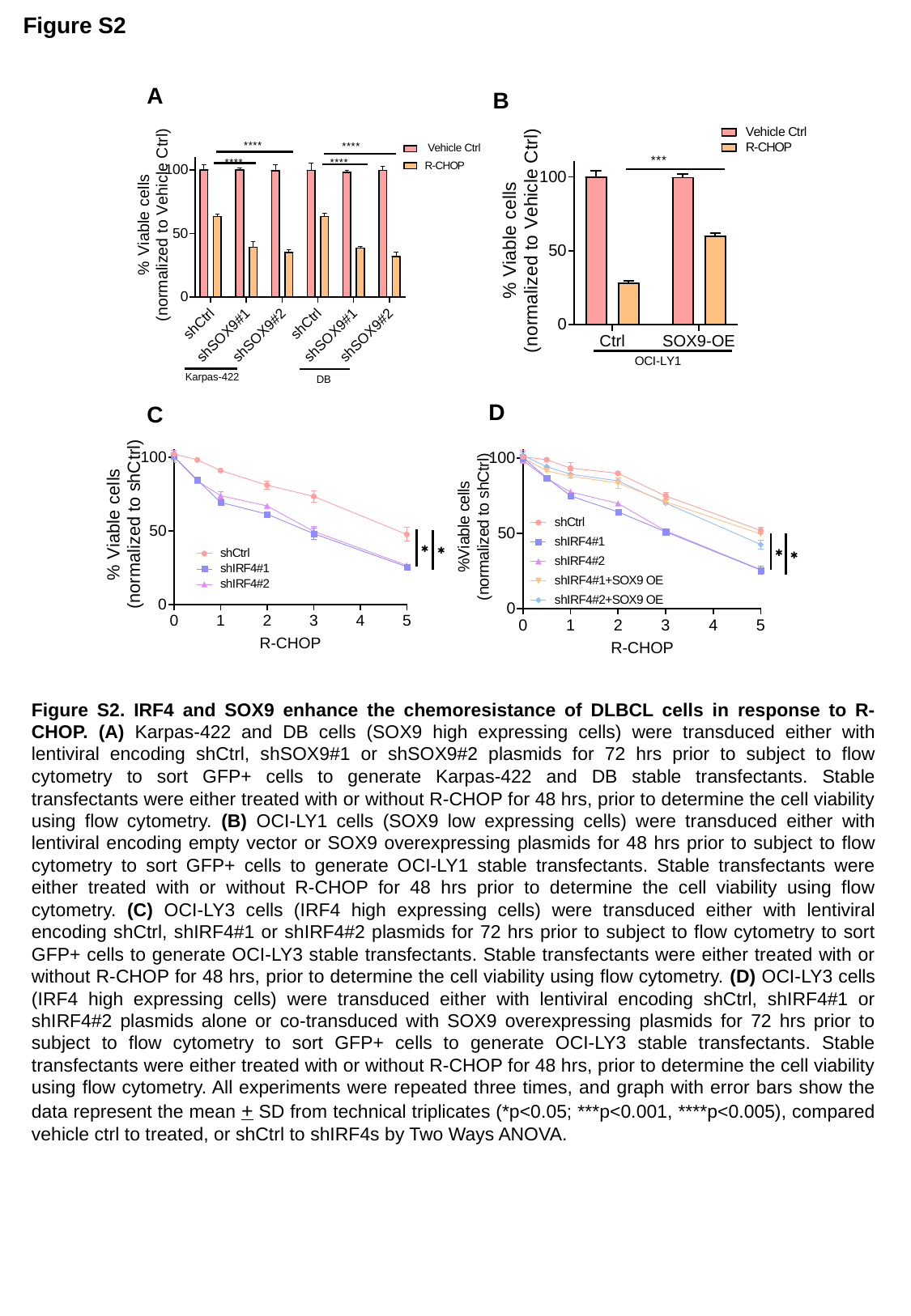

Figure S2
A
B
D
C
Figure S2. IRF4 and SOX9 enhance the chemoresistance of DLBCL cells in response to R-CHOP. (A) Karpas-422 and DB cells (SOX9 high expressing cells) were transduced either with lentiviral encoding shCtrl, shSOX9#1 or shSOX9#2 plasmids for 72 hrs prior to subject to flow cytometry to sort GFP+ cells to generate Karpas-422 and DB stable transfectants. Stable transfectants were either treated with or without R-CHOP for 48 hrs, prior to determine the cell viability using flow cytometry. (B) OCI-LY1 cells (SOX9 low expressing cells) were transduced either with lentiviral encoding empty vector or SOX9 overexpressing plasmids for 48 hrs prior to subject to flow cytometry to sort GFP+ cells to generate OCI-LY1 stable transfectants. Stable transfectants were either treated with or without R-CHOP for 48 hrs prior to determine the cell viability using flow cytometry. (C) OCI-LY3 cells (IRF4 high expressing cells) were transduced either with lentiviral encoding shCtrl, shIRF4#1 or shIRF4#2 plasmids for 72 hrs prior to subject to flow cytometry to sort GFP+ cells to generate OCI-LY3 stable transfectants. Stable transfectants were either treated with or without R-CHOP for 48 hrs, prior to determine the cell viability using flow cytometry. (D) OCI-LY3 cells (IRF4 high expressing cells) were transduced either with lentiviral encoding shCtrl, shIRF4#1 or shIRF4#2 plasmids alone or co-transduced with SOX9 overexpressing plasmids for 72 hrs prior to subject to flow cytometry to sort GFP+ cells to generate OCI-LY3 stable transfectants. Stable transfectants were either treated with or without R-CHOP for 48 hrs, prior to determine the cell viability using flow cytometry. All experiments were repeated three times, and graph with error bars show the data represent the mean + SD from technical triplicates (*p<0.05; ***p<0.001, ****p<0.005), compared vehicle ctrl to treated, or shCtrl to shIRF4s by Two Ways ANOVA.

## Slide 3
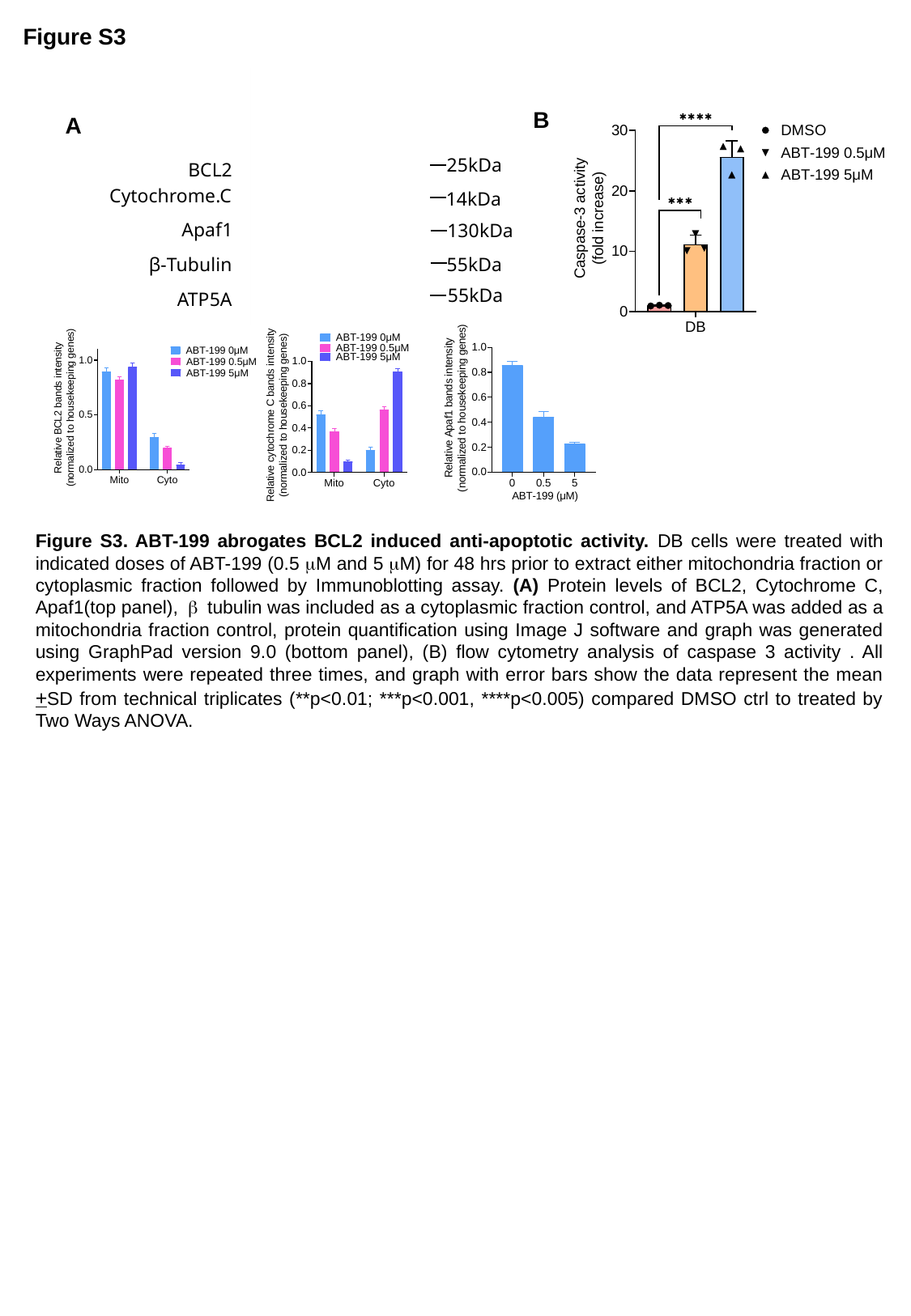

Figure S3
A
BCL2
Cytochrome.C
Apaf1
β-Tubulin
ATP5A
B
25kDa
14kDa
130kDa
55kDa
55kDa
Figure S3. ABT-199 abrogates BCL2 induced anti-apoptotic activity. DB cells were treated with indicated doses of ABT-199 (0.5 mM and 5 mM) for 48 hrs prior to extract either mitochondria fraction or cytoplasmic fraction followed by Immunoblotting assay. (A) Protein levels of BCL2, Cytochrome C, Apaf1(top panel), b tubulin was included as a cytoplasmic fraction control, and ATP5A was added as a mitochondria fraction control, protein quantification using Image J software and graph was generated using GraphPad version 9.0 (bottom panel), (B) flow cytometry analysis of caspase 3 activity . All experiments were repeated three times, and graph with error bars show the data represent the mean +SD from technical triplicates (**p<0.01; ***p<0.001, ****p<0.005) compared DMSO ctrl to treated by Two Ways ANOVA.

## Slide 4
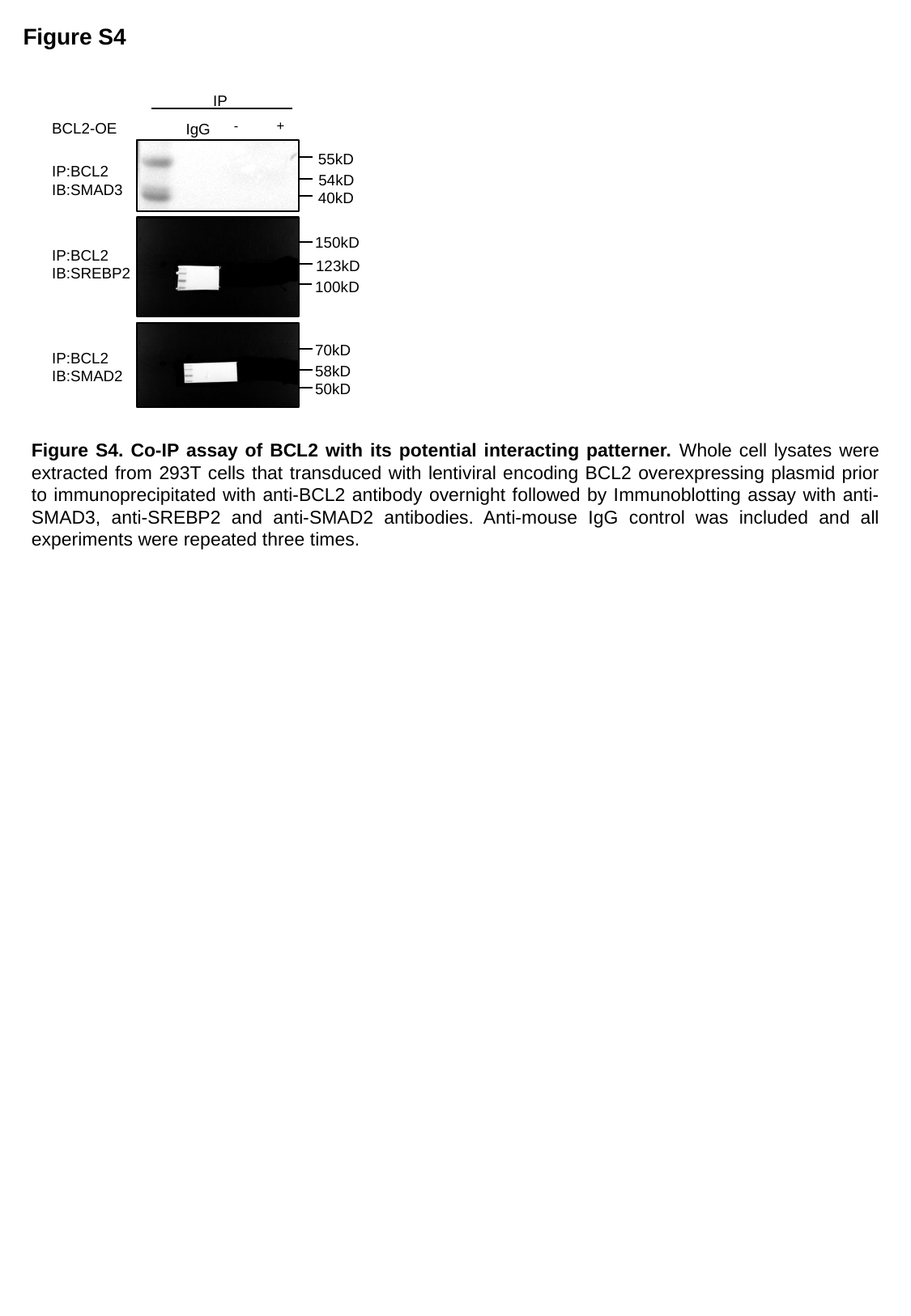

Figure S4
IP
- +
BCL2-OE
IgG
55kD
IP:BCL2
IB:SMAD3
54kD
40kD
150kD
IP:BCL2
IB:SREBP2
123kD
100kD
70kD
IP:BCL2
IB:SMAD2
58kD
50kD
Figure S4. Co-IP assay of BCL2 with its potential interacting patterner. Whole cell lysates were extracted from 293T cells that transduced with lentiviral encoding BCL2 overexpressing plasmid prior to immunoprecipitated with anti-BCL2 antibody overnight followed by Immunoblotting assay with anti-SMAD3, anti-SREBP2 and anti-SMAD2 antibodies. Anti-mouse IgG control was included and all experiments were repeated three times.
