# Supplementary material for: IRF4 contributes to chemoresistance in IGH::BCL2‐positive diffuse large B‐cell lymphomas by mediating BCL2‐induced SOX9 expression
Source: Clin Transl Med. 2025 May 12;15(5):e70336. doi: 10.1002/ctm2.70336 (PMC12069798; doi:10.1002/ctm2.70336)
Supplement: Supplementary file 5 — Supporting Information [file CTM2-15-e70336-s001.docx]

We have included complete blot images in the source data, encompassing crucial details regarding the antibodies and marker positions. The positions of the proteins are demarcated by red boxes. Both the bands corresponding to the target protein(s) and the reference protein (β-tubulin, GAPDH), applied as a loading control, were derived from the same blot. The PVDF membrane underwent segmentation to enable simultaneous detection of the target protein and the reference protein (β-tubulin, GAPDH). However, eventually, we restored the PVDF membrane to its original, unmodified state.

Fig.1A

Fig.1C

Fig.1E

Fig.1F

Fig.3B

IgG	-	+

IP

BCL2-OE

IP:BCL2

IB:SMAD3

50Kd

IP:BCL2

IB:SREBP2

100Kd

IP:BCL2

IB:SMAD2

50Kd

25Kd

Bcl2

Fig.3C

BCL2-OE	-	+	-	+

IRF4 –OE	-	+	+	-

IP:BCL2

IB:IRF4

50Kd

Fig.3D

Fig.3F

Fig.5A

Fig.5B

Fig.5C

shBCL2#2

shBCL2#1

shCtrl

0 6 9 12

0 6 9 12

0 6 9 12

Time (hrs)

+ + +	+

+ + +	+

+ + +	+

CHX(ng/ml)

Fig.5D

+ + +	+

CHX(ng/ml)

shCtrl

ABT-199

Time (hrs)

0 6 9 12

+ + +	+

0 6 9 12

Fig.5E

Fig.S1

Fig.S3

Fig.S4
